# Antitumor Potential of Withanolide Glycosides from Ashwagandha (*Withania somnifera*) on Apoptosis of Human Hepatocellular Carcinoma Cells and Tube Formation in Human Umbilical Vein Endothelial Cells

**DOI:** 10.3390/antiox11091761

**Published:** 2022-09-06

**Authors:** Dahae Lee, Jae Sik Yu, Ji Won Ha, Seoung Rak Lee, Bum Soo Lee, Jin-Chul Kim, Jung Kyu Kim, Ki Sung Kang, Ki Hyun Kim

**Affiliations:** 1 College of Korean Medicine, Gachon University, Seongnam 13120, Korea; 2 School of Pharmacy, Sungkyunkwan University, Suwon 16419, Korea; 3 Department of Integrative Biological Sciences and Industry, Sejong University, Seoul 05006, Korea; 4 KIST Gangneung Institute of Natural Products, Natural Product Informatics Research Center, Gangneung 25451, Korea; 5 School of Chemical Engineering, Sungkyunkwan University, Suwon 16419, Korea

**Keywords:** *Withania somnifera*, withanolide glycosides, hepatocellular carcinoma, HepG2 cells, apoptosis, angiogenesis

## Abstract

Hepatocellular carcinoma (HCC) is the fastest-growing tumor capable of spreading to other organs via blood vessels formed by endothelial cells. Apoptosis and angiogenesis-targeting therapies are attractive for cancer treatment. In this study, we aimed to study the in vitro cytotoxicity of *Withania somnifera* against human HCC (HepG2) cells, identify potential antitumoral withanolide glycosides from the active fraction, and elucidate cytotoxic molecular mechanisms of identified bioactive compounds. *W. somnifera* (Solanaceae), well-known as ‘ashwagandha’, is an Ayurvedic medicinal plant used to promote health and longevity, and the MeOH extract of *W. somnifera* root exhibited cytotoxicity against HepG2 cells during initial screening. Bioactivity-guided fractionation of the MeOH extract and subsequent phytochemical investigation of the active *n*-BuOH-soluble fraction resulted in the isolation of five withanolide glycosides (**1**–**5**), including one new metabolite, withanoside XIII (**1**), aided by liquid chromatography–mass spectrometry-based analysis. The new compound structure was determined by 1D and 2D nuclear magnetic resonance spectroscopy, high-resolution electrospray ionization mass spectroscopy, electronic circular dichroism, and enzymatic hydrolysis. In addition, withanoside XIIIa (**1a**) was identified as the new aglycone (**1a**) of **1**. Isolated withanolide glycosides **1–5** and **1a** were cytotoxic toward HepG2 cells; withagenin A diglucoside (WAD) (**3**) exhibited the most potent cytotoxicity against HepG2 cells, with cell viability less than 50% at 100 μM. WAD cytotoxicity was mediated by both extrinsic and intrinsic apoptosis pathways. Treatment with WAD increased protein expression levels of cleaved caspase-8, cleaved caspase-9, cleaved caspase-3, Bcl-2-associated X protein (Bax), and cleaved poly(ADP-ribose) polymerase (cleaved PARP) but decreased expression levels of B-cell lymphoma 2 (Bcl-2). Moreover, WAD inhibited tubular structure formation in human umbilical vein endothelial cells (HUVECs) by inhibiting the protein expression of vascular endothelial growth factor receptor 2 and its downstream pathways, including extracellular signal-regulated kinase (ERK), phosphoinositide 3-kinase (PI3K), Akt, and mammalian target of rapamycin (mTOR). These effects were also enhanced by co-treatment with ERK and PI3K inhibitors. Overall, these results indicate that WAD (**3**) induced HepG2 apoptosis and inhibited HUVEC tube formation, suggesting its potential application in treating liver cancers.

## 1. Introduction

Hepatocellular carcinoma (HCC) is one of the fastest-growing malignant tumors, capable of spreading to other organs via blood vessels formed by endothelial cells, thereby resulting in extrahepatic and intrahepatic metastases [1]. HCC is associated with high mortality, morbidity, and poor prognosis. Chemotherapy is the standard treatment for HCC [2], and the combination of atezolizumab and bevacizumab is the first-line treatment for advanced HCC [3]; however, the clinical safety of this combination has not yet been fully evaluated in patients [4]. Therefore, there is an urgent need to explore and discover new drugs with fewer toxic side effects to treat patients with HCC. Therapies targeting apoptosis and angiogenesis are attractive strategies for treating cancer [5]. An apoptosis-based therapy that regulates the uncontrolled growth of cancer cells could be a successful nonsurgical treatment option. In addition, therapies targeting angiogenesis are attractive for inhibiting HCC cell metastasis and growth [6].

*Withania somnifera* (L.) Dunal, well-known as ‘ashwagandha’, belongs to the family Solanaceae, and its leaves and roots have been traditionally used to promote longevity and optimal health in Ayurvedic medicine for over 3000 years [7,8]. To date, extracts of *W. sominifera* roots have been widely consumed as functional foods in the form of powder, tablets, and capsules, given their medicinal properties, such as enhanced cognitive ability and stress release. According to previous phytochemical studies, withanolides are the primary bioactive constituents of *W. somnifera* roots and exhibit diverse biological properties, such as neuroprotective, anticancer, anti-inflammatory, immunomodulatory, and antioxidant activities [9,10,11,12,13,14]. More than 40 withanolides, including novel compounds, have been isolated from *W. somnifera* [15], along with some withanolide glycosides with β-D-glucopyranose linked at C-3 or C-27 [15]. In our recent studies on *W. somnifera* roots, we reported the presence of bioactive withanolides, including new compounds, withasilolides A–F [16] and withasomniferol D [17], with some exerting marked cytotoxicity against human cancer cells, as well as anti-adipogenic activity. Few withanolide glycosides have been reported when compared with withanolides, and recently reported withanolide glycosides, withanosides I-XI, were found to display anti-Alzheimer, anti-stress, and neuroprotective activities [18]. Some withanolide glycosides exhibit antiviral activity and could be potential therapeutic agents against coronavirus disease (COVID-19) [18,19]. Based on our recent phytochemical study examining *W. somnifera* roots, withanolide glycosides possess anti-*Helicobacter pylori* and antioxidant effects [20]. Therefore, withanolide glycosides exhibit various promising biological effects that contribute to their health benefits.

As part of a continuing discovery of bioactive phytochemicals with novel structures [21,22,23,24,25], we examined antitumor withanolide glycosides, a relatively unexplored natural product from a methanolic extract of *W. somnifera* roots, using a bioactivity-guided isolation technique. To the best of our knowledge, no previous report has documented the ability of withanolide glycosides to induce apoptosis in human HCC (HepG2) cells or inhibit tube formation in human umbilical vein endothelial cells (HUVECs). Bioactivity-guided fractionation based on cytotoxic activity and subsequent phytochemical investigation of MeOH extracts of *W. somnifera* roots led to the isolation of five withanolide glycosides (**1–5**), including one new metabolite, withanoside XIII (**1**), from the active *n*-BuOH-soluble fraction, which was also aided by liquid chromatography–mass spectrometry (LC/MS)-based analysis. In addition, enzymatic hydrolysis of **1** afforded an aglycone, withanoside XIIIa (**1a**), as a new compound. The structures of newly identified compounds were determined by 1D and 2D nuclear magnetic resonance (NMR) spectroscopy, high-resolution (HR) electrospray ionization (ESI)-MS, and electronic circular dichroism (ECD). We examined the cytotoxicity of isolated withanolide glycosides, including an aglycone of **1**, on HepG2 cell viability and evaluated HUVEC tube formation by selecting the most cytotoxic compound. Herein, we described the bioactivity-guided isolation and structural elucidation of compounds **1–5** and evaluated their antitumor potential and underlying molecular mechanisms.

## 2. Materials and Methods

### 2.1. General Experimental Procedure and Plant Material

Detailed information regarding the general experimental procedure and identification of plant materials is provided in the Supplementary Material.

### 2.2. Extraction and Separation of the Compounds

Dried roots of *W. somnifera* (1.28 kg) were extracted with 80% MeOH for 3 days under reflux. The detained procedure for the isolation of compounds **1–5** from the MeOH extract is provided in the Supplementary Material.

#### Withanoside XIII (**1**)

White amorphous powder; [α]${}_{D}^{25}$−12.3 (*c* 0.28, MeOH); UV (MeOH) λ_max_ (log ε) 202 (2.8) nm; IR (KBr) ν_max_ 3421, 2969, 1679, 1657, 1369, 1025 cm^–1^; ECD (MeOH) λ_max_ (Δε) 216 (+0.31), 254 (+0.71) nm; ^1^H (850 MHz) and ^13^C NMR (212.5 MHz), see Table S1; (−)− ESI-MS *m*/*z* 843 [M + HCOO]^−^; (−)− HR-ESIMS *m*/*z* 843.3948 [M + HCOO]^−^ (calcd. for C_41_H_63_O_18_, 843.4014).

### 2.3. Enzymatic Hydrolysis and Absolute Configuration Determination of Sugar Moieties of Compound **1**

The absolute configuration of sugar moieties in **1** was determined using a previously described method [26]. Briefly, compound **1** (1.5 mg) was hydrolyzed with crude glucosidase (10 mg, from almonds, Sigma-Aldrich, Saint Louis, MO, USA) for 72 h at 37 °C, and MC was used for aglycone extraction. LC/MS analysis of the MC fraction confirmed the presence of aglycone **1a**. A detailed description of the absolute configuration of the sugar moieties in the aqueous layer is included in the Supplementary Materials.

#### Withanoside XIIIa (**1a**)

White amorphous powder; [α]${}_{D}^{25}$−8.0 (*c* 0.07, MeOH); UV (MeOH) λ_max_ (log ε) 200 (2.5) nm; IR (KBr) ν_max_ 3441 2965, 1681, 1654, 1367, 1022 cm^−1^; ECD (MeOH) λ_max_ (Δε) 212 (+1.95), 255 (+1.65) nm; ^1^H (850 MHz) and ^13^C NMR (212.5 MHz), see Table S1; (−)− ESI-MS *m*/*z* 519 [M + HCOO]^−^; (−)− HR-ESI-MS *m*/*z* 519.2947 [M + HCOO]^−^ (calcd. for C_2__9_H_4__3_O_8_, 519.2958).

### 2.4. Cell Culture

The HepG2 HCC cells (HB-8065) were obtained from American Type Culture Collection Manassas (ATCC; Manassas, VA, USA), maintained in a Dulbecco’s modified Eagle medium (Cellgro, Manassas, VA, USA), supplemented with 10% fetal bovine serum (Gibco BRL, Carlsbad, MD, USA) and 1% penicillin/streptomycin (P/S; Gibco BRL, Carlsbad, MD, USA). HUVECs (CRL-1730) were obtained from ATCC and maintained using Clonetics EGM-2 MV BulletKit (Takara Bio Inc., Shiga, Japan). HepG2 cells and HUVECs seeded in a 100 mm dish were incubated at 37 °C in incubators (humidified atmosphere with 5% CO_2_ and 95% air).

### 2.5. Cell Viability Assay

The culture medium containing HepG2 cells (1 × 10^4^ cells per well) and HUVECs (5 × 10^5^ cells per well) were seeded in 96-well culture plates and cultivated for 24 h. Extracts and compounds were dissolved in dimethyl sulfoxide (Sigma-Aldrich, Saint Louis, MO, USA) to yield 100 mg/mL or 100 mM stocks. The stocks were aliquoted and kept at −80 °C until use. Required concentrations of each extract and compound were made by dilutions with culture media. Cells were treated with a culture medium containing varying concentrations of extracts, fractions, and compounds by serial dilution for 24 h. Cells were treated with culture media containing 0.5% DMSO as vehicle controls. Subsequently, Ez-Cytox reagent (Daeil Lab Service Co., Seoul, Korea) was added to each well. After incubation for 1 h, the absorbance was recorded at 490 nm using a PowerWave XS microplate reader (Bio-Tek Instruments, Winooski, VT, USA) to quantify the cell viability.

### 2.6. Tube Formation Assay

Briefly, 96-well culture plates were coated with 10 mg/mL Matrigel (BD Biosciences, San Jose, CA, USA) at 37 °C for 30 min. After coating, HUVECs were plated at a density of 5 × 10^5^ cells per well in a medium containing WAD (25, 50, and 100 μM) for 24 h. A 4% paraformaldehyde solution (Sigma Aldrich, St. Louis, MO, USA) was added to each well before adding hematoxylin (Muto Pure Chemicals, Tokyo, Japan). Tubular structure formation in HUVECs was stained with hematoxylin and inspected using a phase-contrast microscope at 200 × magnification. Tube length was quantified using the ImageJ software (version 1.48; National Institutes of Health, Bethesda, MD, USA). Data are expressed as the percentage increase in the total tube length of HUVECs.

### 2.7. Western Blotting Analysis

Following treatment, equal amounts of cellular lysates were separated on 10% sodium dodecyl sulfate-polyacrylamide gel, transferred to polyvinylidene difluoride membranes (Pall Corporation, Washington, NY, USA), and probed with 1:1000 dilution of primary antibodies (Cell Signaling, Boston, MA, USA) overnight at 4 °C. The appropriate secondary antibody (Cell Signaling, Boston, MA, USA) was used to detect the primary antibodies. Immune complexes were analyzed using a chemiluminescence system (FUSION Solo; PEQLAB Biotechnologie GmbH, Erlangen, Germany).

### 2.8. Statistical Analysis

Statistical analysis was performed using one-way analysis of variance and multiple comparisons with Bonferroni correction. Statistical significance was set at *p* < 0.05. Data analyses were performed using the SPSS Statistics ver. 19.0 (SPSS Inc., Chicago, IL, USA).

## 3. Results

### 3.1. Bioactivity-Guided Isolation of Compounds **1–5**

**Figure 1:**
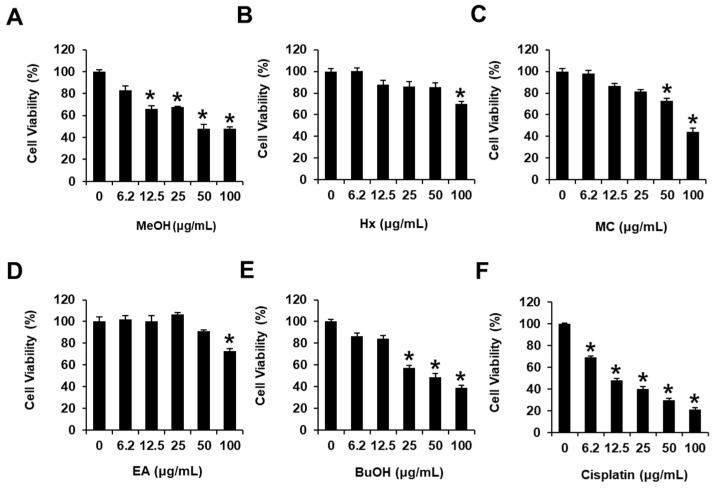
Effects of the MeOH extract of *Withania somnifera* roots and hexane (Hx), dichloromethane (MC), ethyl acetate (EA), and *n*-butanol (BuOH)-soluble fractions on human hepatocellular carcinoma (HepG2) cell viability. HepG2 cells were treated with (**A**) MeOH extract (MeOH), (**B**) Hx, (**C**) MC, (**D**) EA, (**E**) BuOH-soluble fractions, and (**F**) cisplatin (6.2, 12.5, 25, 50 and 100 μg/mL) for 24 h. Cells were treated with culture media containing 0.5% DMSO as vehicle controls. The MTT assay assessed HepG2 cell viability. Data are presented as the mean ± standard error of the mean (SEM). *n* = 3, * *p* < 0.05 compared with the control.

The MeOH extract of *W. somnifera* roots exhibited cytotoxicity toward human HCC cells, i.e., HepG2 cells, reducing the cell viability to 69.88 ± 2.21% at 100 μg/mL (Figure 1). The MeOH extract was sequentially subjected to the solvent partition process using four solvents, hexane (Hx), dichloromethane (MC), ethyl acetate (EA), and *n*-butanol (BuOH), to yield four main fractions; the cytotoxicity of these fractions was examined in HepG2 cells to determine bioactive compounds in the MeOH extract (Figure 1). Assessment of viable cells using the MTT assay revealed that the BuOH-soluble fraction significantly reduced cell viability in HepG2 cells in a dose-dependent manner, exhibiting cell viabilities of 57.25 ± 2.63, 48.67 ± 3.28, and 39.32 ± 2.28% at 25, 50, and 100 μg/mL, respectively (Figure 1); this fraction was deemed the most active faction from among the four main fractions. These findings suggested that the MeOH extract of *W. somnifera* roots exerted cytotoxicity against HepG2 cells, and the BuOH-soluble fraction contained bioactive constituent(s) mediating the observed cytotoxicity.

**Figure 2:**
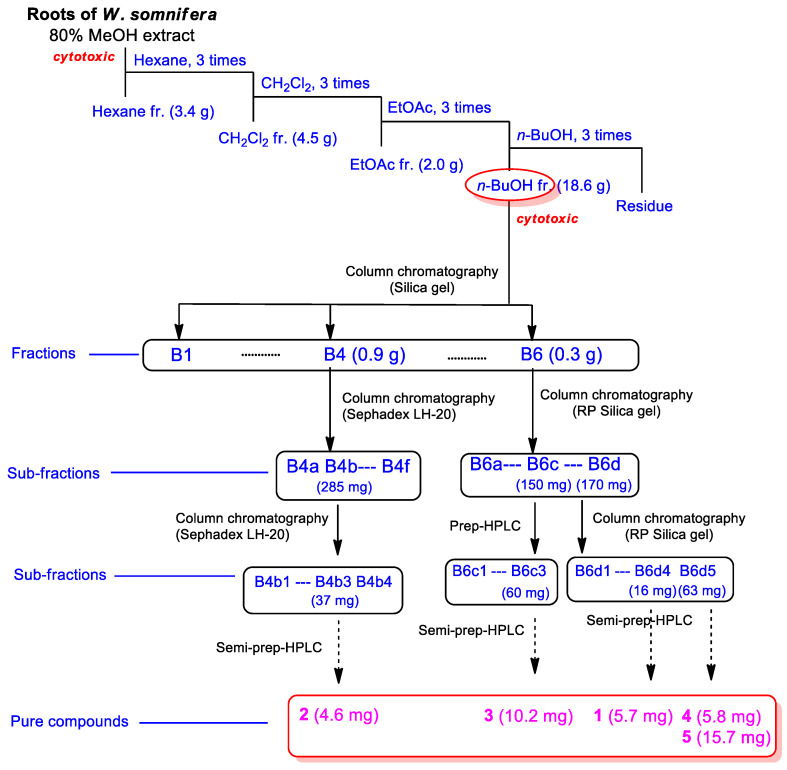
Bioactivity-guided isolation of compounds **1**–**5**.

Accordingly, we examined the BuOH fraction to identify constituents mediating in vitro cytotoxicity against HepG2 cells. Chemical assessment of the active BuOH fraction using repeated column chromatography and semi-preparative high-performance liquid chromatography (HPLC), along with LC/MS analysis using an in-house-built UV library database, resulted in the isolation of five withanolide glycosides (**1–5**) (Figure 2).

### 3.2. Structural Elucidation of Isolated Compounds **1–5**

**Figure 3:**
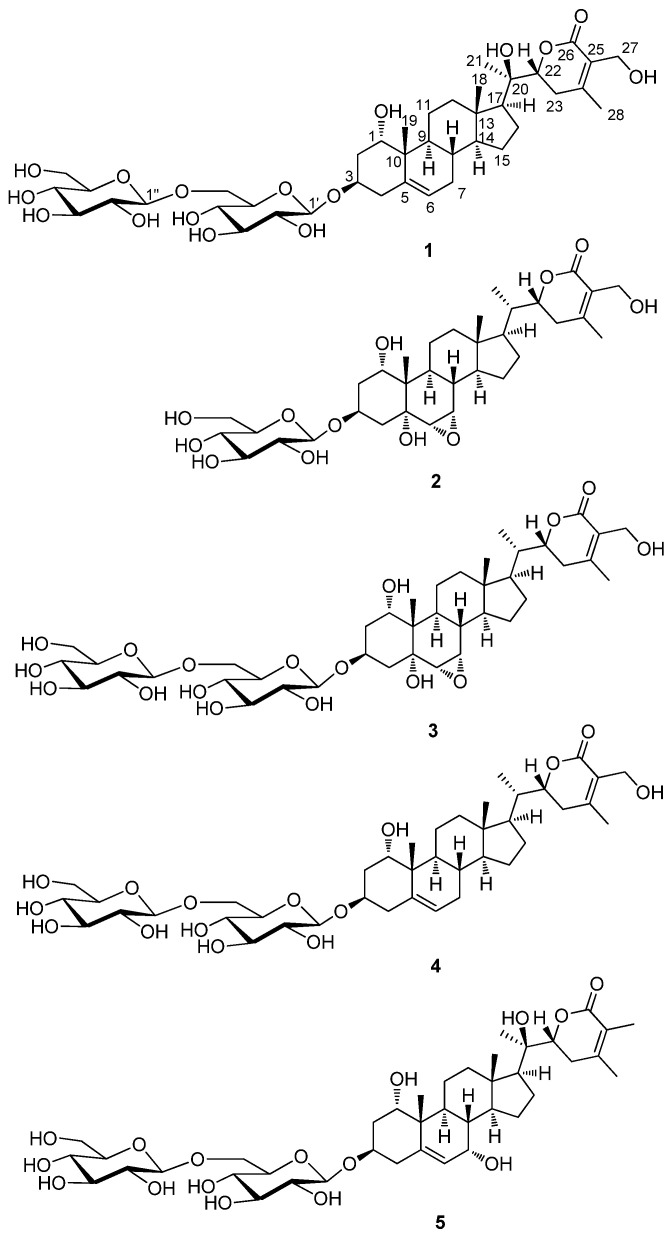
Chemical structures of compounds **1**–**5**.

Compound **1**, isolated as a white amorphous powder, exhibited the molecular formula C_40_H_62_O_16_, which was detected by the negative-ion mode of HR-ESIMS, revealing an [M + HCOO]^−^ ion peak at *m/z* 843.3948 (calcd. for C_41_H_63_O_18_, 843.4014). The infrared (IR) spectrum of **1** showed characteristic absorptions for hydroxy (3421 cm^−1^) and α,β-unsaturated ketone (1679 cm^−1^) functional groups. ^1^H NMR data (Table S1) of compound **1**, assigned by the aid of heteronuclear single quantum correlation (HSQC) experiment, indicated the presence of signals for four methyls (δ_H_ 0.88 (3H, s), 1.02 (3H, s), 1.27 (3H, s), and 2.11 (3H, s)), three oxygenated methines (δ_H_ 3.81 (1H, dd, *J* = 1.5, 1.5 Hz), 4.03 (1H, dddd, *J* = 12.5, 12.5, 5.5, 5.5 Hz), and 4.29 (1H, dd, *J* = 13.0, 3.5 Hz)), one oxygenated methylene (δ_H_ 4.32 (1H, d, *J* = 12.0 Hz) and 4.40 (1H, d, *J* = 12.0 Hz)), and one olefinic proton (δ_H_ 5.53 (1H, d, *J* = 5.5 Hz)), along with two indicative anomeric protons (δ_H_ 4.37 (1H, d, *J* = 8.0 Hz), 4.39 (1H, d, *J* = 8.0 Hz)) attributable to sugar moieties. ^13^C NMR data (Table S1) of **1**, combined with heteronuclear multiple bond correlation (HMBC) experiments, revealed 40 carbon resonances, including 28 carbons for aglycone and 12 carbons for sugar units. A detailed inspection of NMR data demonstrated that NMR data of **1** were markedly similar to those of withanoside IV [27], also isolated as compound **4** in the present study (Figure 3); however, apparent differences between compounds **1** and **4** were observed at C-20 owing to the discrepancy in NMR data corresponding to C-20 and C-21.

**Figure 4:**
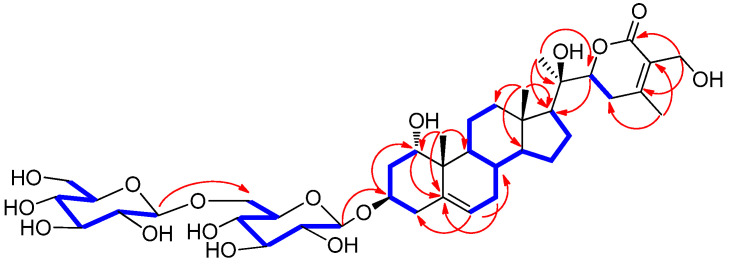
Key ^1^H-^1^H COSY (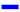) and HMBC (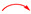) correlations for **1**.

The differential partial structure of **1** was determined and confirmed by analysis of the key HMBC correlations of H_3_-21 (δ_H_ 1.27)/C-17 (δ_C_ 54.4), C-20 (δ_C_ 73.3), and C-22 (δ_C_ 81.4) (Figure 4). Furthermore, the linkage of the two glucose moieties was verified by the key COSY correlations from H-1 to H_2_-4 and key HMBC correlations of H-1′‘/C-6′ and H-1′/C-3 (Figure 4). The complete gross structure of **1** was elucidated by undertaking a detailed interpretation of the COSY and HMBC correlations (Figure 4).

**Figure 5:**
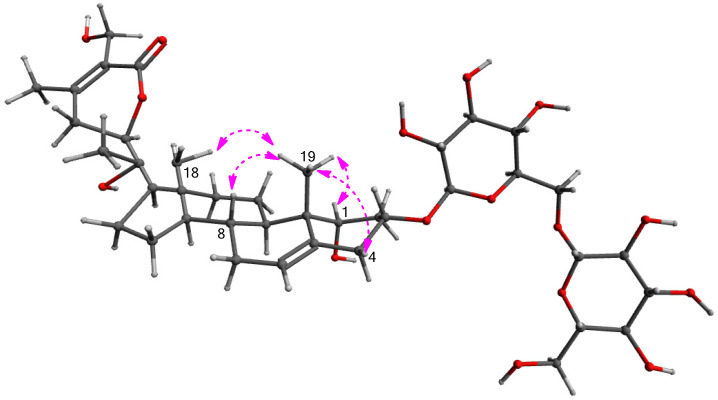
Key ROESY (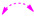) correlations for **1**.

The stereochemistry of **1** was determined by analyzing vicinal proton coupling constants observed in ^1^H NMR, the correlations from the rotating frame Overhauser effect spectroscopy (ROESY) experiment, and ECD data. The α-position of the hydroxy group on C-1 was established by ROESY correlations of H_3_-19/H-1, H-4β, H-8/H_3_-18, and H_3_-19 (Figure 5). The ECD spectrum of **1** showed a positive Cotton effect at 254 nm due to the n → π* transition of the *α*,β-unsaturated δ-lactone [28], indicating a 22R-configuration. The configuration of C-22 was also supported by characteristic coupling constants (*J* = 13.0 and 3.5 Hz) of H-22, presenting a doublet of doublets [28]. The configuration of C-20 of **1** was found to be similar to that of related compounds, withasilolide A [16], 20β-hydroxy-1-oxo-(22R)-witha-2,5,24-trienolide [29], dunawithanine B [30], and withacoagulin E [31] with comparable ^13^C NMR chemical shifts observed for C-20. In addition, the enzymatic hydrolysis of **1** using glucosidase yielded aglycone **1a** and sugar moieties. The absolute configuration of the two glucose moieties of **1** was confirmed to be D-configuration by LC/MS-based analysis following thiocarbamoyl-thiazolidine derivatization [26]; this also demonstrated that both sugar units of **1** are β-D-glucopyranoses, given the typical coupling constant (*J* = 8.0 Hz) of anomeric protons, which is characteristic of β-form in glucopyranose [32]. Therefore, the chemical structure of **1** was determined, as shown in Figure 3, and compound **1** was designated withanoside XIII.

**Figure 6:**
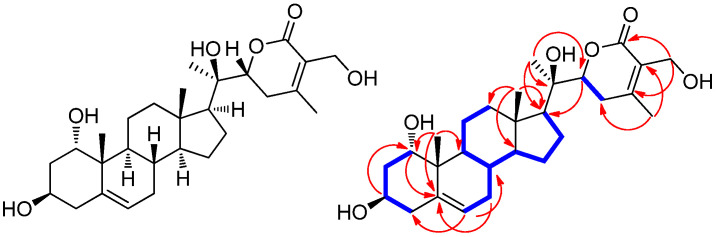
Structure of **1a** and key ^1^H-^1^H COSY (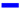) and HMBC (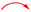) correlations for **1a**.

Compound **1a**, derived as an aglycone of **1** by enzymatic hydrolysis using glucosidase, presented the molecular formula C_28_H_42_O_6_ based on HR-ESIMS, which showed a molecular ion peak at *m*/*z* 519.2947 [M + HCOO]^−^ (calcd. for C_29_H_43_O_8_, 519.2958) in negative-ion mode. NMR data of **1a** revealed resonances similar to those of compound **1**, in addition to the corresponding signals for sugar moieties. The up-field shifted C-3 chemical shifts (δ_H_ 3.89/δ_C_ 65.7) of **1a** supported the removal of sugar units at C-3. In addition, the gross planar structure of **1a** was established by interpreting the correlations observed in COSY and HMBC experiments (Figure 6). The identical ROESY correlations and similar Cotton effects in the ECD spectrum of **1a** and **1** confirmed that **1a** exhibited a similar stereochemistry to that **1**. Accordingly, the structure of **1a** was determined, as shown in Figure 6, which was identified as a new compound, subsequently named withanoside XIIIa.

Other known compounds were identified as withanoside III (**2**) [27], withagenin A diglucoside (**3**) (WAD) [20,33], withanoside IV (**4**) [27], and withanoside XII (**4**) [20] by comparing NMR spectroscopic data with those previously reported and MS data from LC/MS analysis.

### 3.3. Effects of the Isolated Compounds **1–5** on Cell Viability of HepG2 Cells

**Figure 7:**
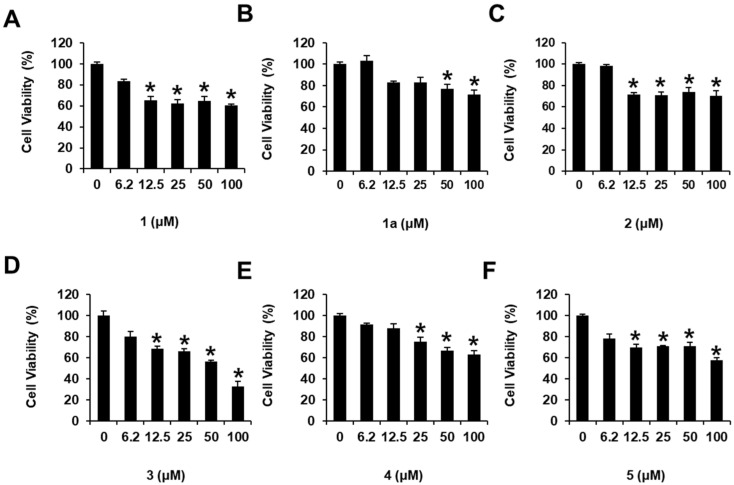
Effects of compounds **1–5** and **1a** on human hepatocellular carcinoma (HepG2) cell viability. HepG2 cells were treated with compounds **1** (**A**), **1a** (**B**), **2** (**C**), **3** (**D**), **4** (**E**), and **5** (**F**) (6.2, 12.5, 25, 50, and 100 μM) for 24 h. The HepG2 cell viability was assessed using the Ez-Cytox cell viability assay. Cells were treated with culture media containing 0.5% DMSO as vehicle controls. Data are presented as the mean ± standard error of the mean (SEM). *n* = 3, * *p* < 0.05 compared with the control.

We next determined whether compounds **1–5** isolated from the cytotoxic fraction, as well as compound **1a**, mediated cytotoxicity against HepG2 cells in vitro. Accordingly, we examined the effects of compounds **1–5** and **1a** on HepG2 cell viability (Figure 7) using the Ez-Cytox cell viability assay. Compounds **1–5** and **1a** exerted cytotoxicity against HepG2 cells, reducing cell viability (60.09 ± 1.76% for **1**, 71.62 ± 4.25% for **1a**, 70.61 ± 4.73% for **2**, 33.01 ± 4.61% for **3**, 63.33 ± 3.38% for **4**, and 57.73 ± 3.66% for **5**) at 100 μM (Figure 7). Among isolated compounds, WAD (**3**) exhibited the highest cytotoxicity against HepG2 cells, which was dose-dependent (Figure 7). These findings suggested that all isolated compounds, at least to some extent, contributed to the cytotoxicity induced by *W. somnifera* roots against HCC cells in vitro. Moreover, WAD (**3**), the most active compound, was selected for the following experiments.

### 3.4. Effects of WAD (**3**) on Apoptosis Signaling Pathways in HepG2 Cells

**Figure 8:**
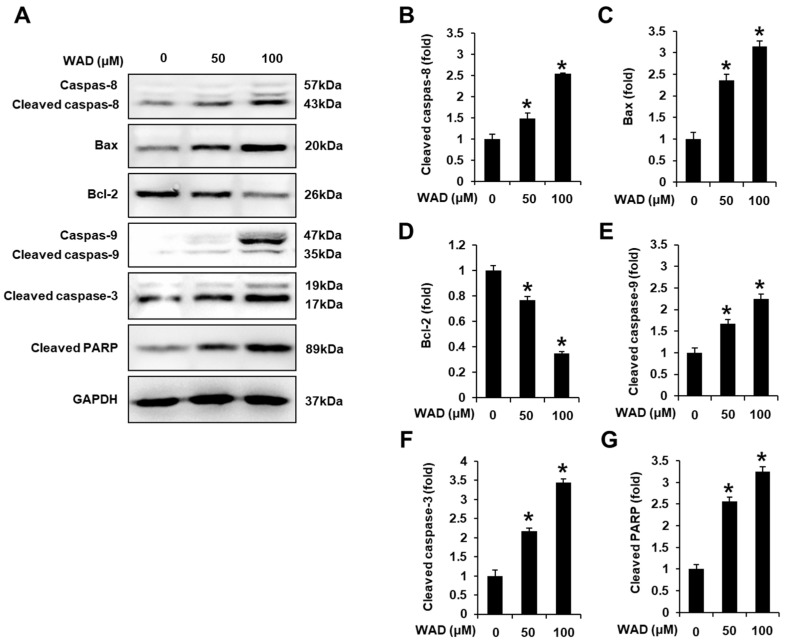
Effects of withagenin A diglucoside (WAD) (**3**) on apoptosis signaling pathways in human hepatocellular carcinoma (HepG2) cells. HepG2 cells were treated with WAD (50 and 100 μM) for 24 h. Cells were treated with culture media containing 0.5% DMSO as vehicle controls: (**A**) Protein expression levels of cleaved caspase-8, Bcl-2-associated X protein (Bax), B-cell lymphoma 2 (Bcl-2), cleaved caspase-9, cleaved caspase-3, cleaved poly(ADP-ribose) polymerase (cleaved PARP), and glyceraldehyde-3-phosphate dehydrogenase (GAPDH). (**B**–**G**) Each bar graph presents the densitometric quantification of protein expression levels. Data are presented as the mean ± standard error of the mean (SEM). *n* = 3, * *p* < 0.05 compared with the control.

Next, we performed Western blotting to determine protein expression levels and evaluate the potential molecular mechanisms of WAD (**3**) on apoptosis in HepG2 cells. Treatment with WAD (50 and 100 μM) increased the protein expression levels of cleaved caspase-8, Bcl-2-associated X protein (Bax), cleaved caspase-9, cleaved caspase-3, and cleaved poly(ADP-ribose) polymerase (cleaved PARP) while decreasing those of B-cell lymphoma 2 (Bcl-2) (Figure 8).

### 3.5. Effects of WAD (**3**) on HUVEC Tube Formation

**Figure 9:**
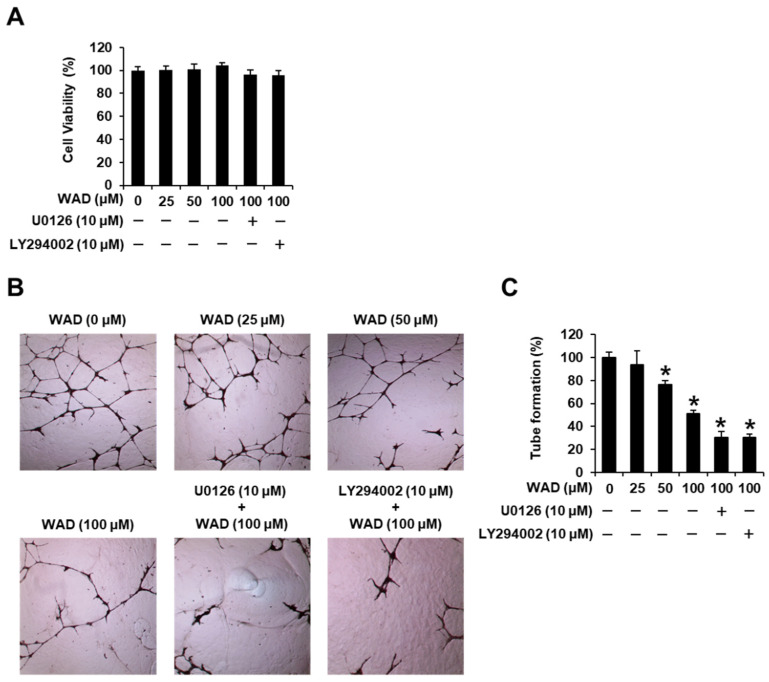
Effects of withagenin A diglucoside (WAD) on cell viability and tube formation in human umbilical vein endothelial cells (HUVECs). HUVECs were treated with WAD (25, 50, and 100 μM), U0126 (10 μM), and/or LY294002 (10 μM) for 24 h. Cells were treated with culture media containing 0.5% DMSO as vehicle controls: (**A**) The MTT assay assessed the viability of HUVECs. (**B**) Representative photographs of HUVEC tube formation on matrigel at 200× magnification. (**C**) The relative lengths of tubes were quantified using ImageJ software. Data are presented as the mean ± standard error of the mean (SEM). *n* = 3, * *p* < 0.05 compared with the control.

Initially, a cell viability assay was performed to evaluate the non-toxic concentrations of WAD in HUVECs, treated with WAD (25, 50, and 100 μM), U0126 (10 μM), and/or LY294002 (10 μM) for 24 h. HUVEC viability was assessed using the MTT assay. Treatment with WAD (25, 50, and 100 μM), U0126 (10 μM), and LY294002 (10 μM) did not affect HUVEC viability (Figure 9A). These non-toxic concentrations were employed to evaluate tube formation in HUVECs. On incubating HUVECs with WAD (25, 50, and 100 μM), tube formation was reduced by 76.38 ± 3.41 and 51.36 ± 2.75% at 50 μM and 100 μM, respectively. These effects were enhanced following co-treatment with a mitogen-activated protein kinase (MEK)/extracellular signal-regulated kinase (ERK) inhibitor (U0126) or a phosphatidylinositol 3-kinase (PI3K) inhibitor (LY294002). Incubation of HUVECs with 100 μM WAD and 10 μM U0126 reduced tube formation by 30.58 ± 4.81%. Treatment with 100 μM WAD and 10 μM LY294002 reduced tube formation by 30.48 ± 2.76% (Figure 9B,C).

### 3.6. Effects of WAD (**3**) on the Vascular Endothelial Growth Factor Receptor (VEGFR) 2 Signaling Pathway in HUVECs

**Figure 10:**
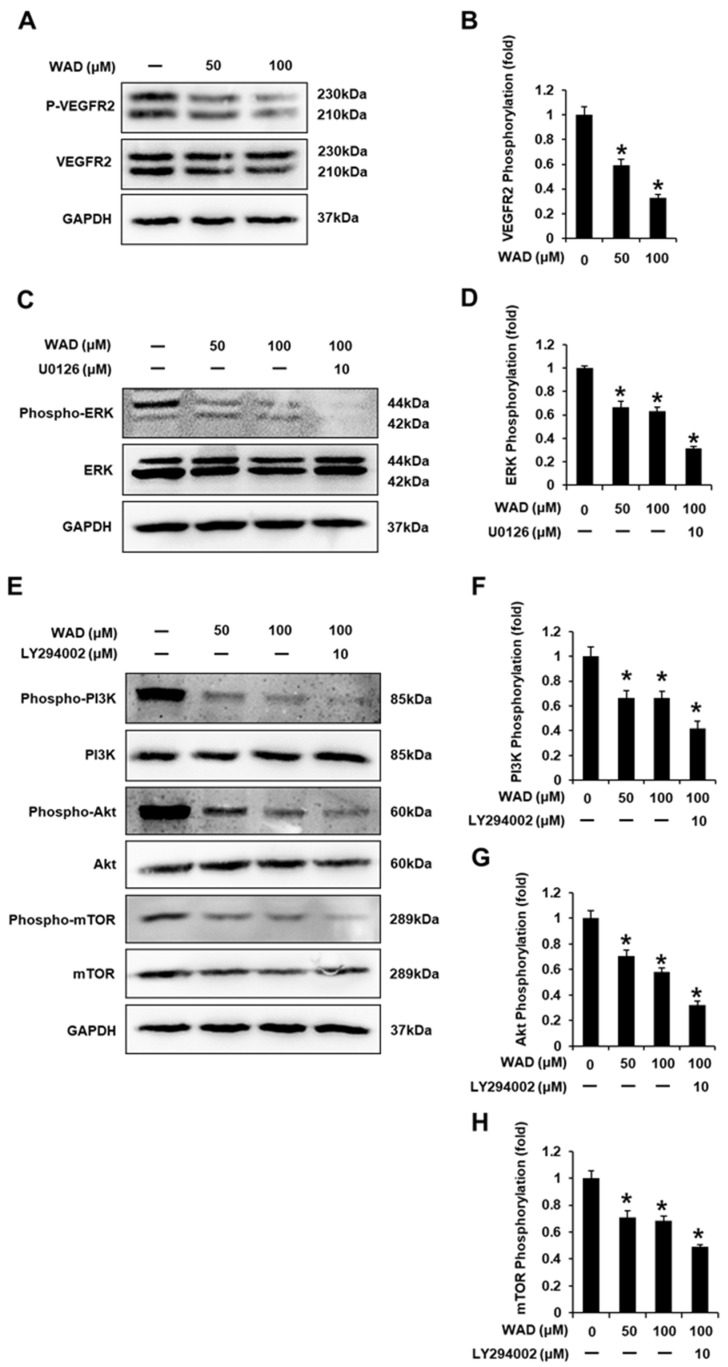
Effects of withagenin A diglucoside (WAD) on the vascular endothelial growth factor receptor 2 (VEGFR2) signaling pathway in human umbilical vein endothelial cells (HUVECs). HUVECs were treated with WAD (50 and 100 μM), U0126 (10 μM), and/or LY294002 (10 μM) for 24 h. Cells were treated with culture media containing 0.5% DMSO as vehicle controls. Protein expression levels of (**A**,**B**) phospho-VEGFR2), VEGFR2, (**C**,**D**) phospho-extracellular signal-regulated kinase (Phospho-ERK), ERK, (**E**–**H**) phospho-phosphoinositide 3-kinase (Phospho-PI3K), PI3K, phospho-Akt, Akt, phospho-mammalian target of rapamycin (Phospho-mTOR), mTOR, and glyceraldehyde-3-phosphate dehydrogenase (GAPDH). Each bar graph presents the densitometric quantification of protein expression levels. Data are presented as the mean ± standard error of the mean (SEM). *n* = 3, * *p* < 0.05 compared with the control.

Western blotting was performed to determine protein expression levels and evaluate the potential molecular mechanisms of WAD on HUVEC tube formation. Treatment with WAD (50 and 100 μM) decreased VEGFR2 phosphorylation (Figure 10A,B). In addition, treatment with WAD (50 and 100 μM) reduced ERK phosphorylation. This effect was enhanced by co-treatment with 10 μM U0126 (Figure 10C,D). Treatment with WAD (50 and 100 μM) decreased the phosphorylation of phosphoinositide 3-kinase (PI3K), Akt, and mammalian target of rapamycin (mTOR), which was enhanced by co-treatment with 10 μM LY294002 (Figure 10E–H). These results indicated the potential mechanism of action of WAD on the angiogenesis of HUVECs.

## 4. Discussion

Insufficient apoptosis has been associated with the development of HCC. Apoptosis is a genetically programmed process leading to cell death that occurs via the activation of extrinsic or intrinsic apoptosis pathways [34]. Herein, the cell viability results were consistent with changes in expression levels of apoptosis-related proteins. The incubation of HepG2 cells with 100 μM WAD reduced cell viability to less than 50%. Following the activation of death receptors in the extrinsic apoptosis pathway, WAD increased the activation of caspase-8. Cleaved caspase-8 activates the final effector of apoptosis, caspase-3 [35]. In addition, treatment with WAD could increase the expression of the pro-apoptotic Bax protein, along with a concomitant reduction in anti-apoptotic Bcl-2 protein expression. Expression patterns of Bax and Bcl-2 induce the release of cytochrome c, which promotes caspase-9 activation [36]. Treatment with WAD induced the intrinsic apoptosis pathway via caspase-9 activation, resulting in altered levels of caspase-3 and cleaved PARP. In the final stage of apoptosis, cleaved PARP induces DNA damage [37]. Accordingly, our results indicated that WAD-induced inhibition of HepG2 cell viability was associated with the induction of both extrinsic and intrinsic apoptosis pathways.

**Figure 11:**
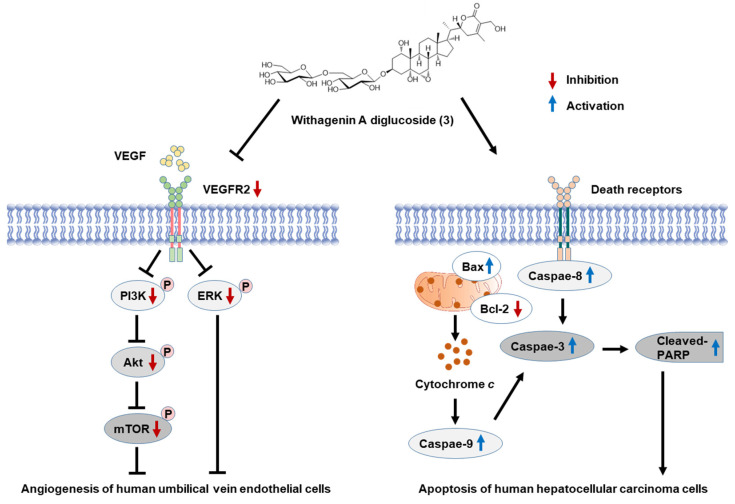
Schematic pathway illustrating the potential role of withagenin A diglucoside (WAD) on apoptosis of human hepatocellular carcinoma cells and tube formation in human umbilical vein endothelial cells. VEGFR2, vascular endothelial growth factor receptor 2.

Tube formation of vascular endothelial cells to grow new blood vessels, namely, angiogenesis, is a vital process during extrahepatic and intrahepatic metastases [38]. Angiogenesis-targeting therapies are an attractive strategy for cancer therapy. In the present study, we observed that WAD inhibited the formation of capillary tube-like structures in HUVECs. This effect was also enhanced following co-treatment with ERK and PI3K inhibitors, respectively, consistent with protein expression levels. Treatment with WAD reduced the phosphorylation of VEGFR2, a crucial angiogenic stimulator during tumor neovascularization [39]. VEGFR2 functions upstream to activate ERK, PI3K, Akt, and mTOR [40]. Treatment with WAD decreased the phosphorylation of ERK, PI3K, Akt, and mTOR. ERK is specifically phosphorylated and activated during vascular development in sprouting vascular endothelial cells [41]. Activated PI3K/AKT elevates the phosphorylation of mTOR to induce endothelial cell proliferation and new blood vessel formation [42]. Collectively, our results indicate that WAD suppressed HUVEC tubular structure formation by inhibiting VEGFR2 and its downstream pathways, including ERK, PI3K, Akt, and mTOR. Accordingly, the findings of the present study may afford a better understanding of mechanisms underlying the anti-angiogenic and apoptotic properties of WAD (Figure 11). Further studies are required to clarify the anticancer activity of WAD using an HepG2 xenograft mouse model and an angiogenic animal model.

## 5. Conclusions

In conclusion, bioactivity-guided fractionation of the MeOH extract of *W. somnifera* roots and phytochemical investigation of the active *n*-BuOH-soluble fraction resulted in the isolation of five withanolide glycosides (**1–5**), including a new compound, withanoside XIII (**1**), via LC/MS-based analysis. The structure of withanoside XIII was determined based on 1D and 2D NMR spectroscopic, HR-ESIMS, and ECD data, along with enzymatic hydrolysis, which afforded a new compound, withanoside XIIIa (**1a**), an aglycone of **1**. Among isolated compounds, WAD (**3**) exhibited the most potent cytotoxicity against human HCC cells (HepG2), which was mediated by both extrinsic and intrinsic apoptosis pathways. Treatment with WAD increased the protein expression levels of cleaved caspase-8, Bax, cleaved caspase-9, cleaved caspase-3, and PARP but decreased Bcl-2 expression. In addition, WAD inhibited tubular structure formation of HUVECs by suppressing VEGFR2 and its downstream pathways, including ERK, PI3K, Akt, and mTOR, thereby elucidating the mechanisms underlying its anti-angiogenic effect. These findings provide experimental evidence for the antitumor potential of WAD (**3**) on anti-angiogenic and apoptotic properties in human HCC cells, suggesting its potential application in treating liver cancers.

## Data Availability

Data is contained within the article and supplementary material.

## Supplementary Materials

The following supporting information can be downloaded at: https://www.mdpi.com/article/10.3390/antiox11091761/s1, Figure S1: HR-ESIMS data of **1**; Figure S2: ^1^H NMR spectrum of **1** (CD_3_OD, 850 MHz); Figure S3: ^13^C NMR spectrum of **1** (CD_3_OD, 212.5 MHz); Figure S4: ^1^H-^1^H COSY spectrum of **1** (CD_3_OD); Figure S5: HSQC spectrum of **1** (CD_3_OD); Figure S6: HMBC spectrum of **1** (CD_3_OD); Figure S7: ROESY spectrum of **1** (CD_3_OD); Figure S8: HR-ESIMS data of **1a**; Figure S9: ^1^H NMR spectrum of **1a** (CD_3_OD, 850 MHz); Figure S10: ^13^C NMR spectrum of **1a** (CD_3_OD, 212.5 MHz); Figure S11: ^1^H-^1^H COSY spectrum of **1a** (CD_3_OD); Figure S12: HSQC spectrum of **1a** (CD_3_OD); Figure S13: HMBC spectrum of **1a** (CD_3_OD); Figure S14: ROESY spectrum of **1a** (CD_3_OD); General experimental procedure; Table S1; ^1^H (850 MHz) and ^13^C NMR (212.5 MHz) data for compounds **1** and **1a** in CD_3_OD (δ ppm).
