# Supplementary material for: Antitumor Potential of Withanolide Glycosides from Ashwagandha (Withania somnifera) on Apoptosis of Human Hepatocellular Carcinoma Cells and Tube Formation in Human Umbilical Vein Endothelial Cells
Source: Antioxidants (Basel). 2022 Sep 6;11(9):1761. doi: 10.3390/antiox11091761 (PMC9495654; doi:10.3390/antiox11091761)
Supplement: Supplementary file 1 [file antioxidants-11-01761-s001.zip › antioxidants-1851662-supplementary.pdf]

Supplementary data

Antitumor Potential of Withanolide Glycosides from Ashwagandha (*Withania somnifera*) on Apoptosis of Human Hepatocellular Carcinoma Cells and Tube Formation in Human Umbilical Vein Endothelial Cells

Dahae Lee [1,†], Jae Sik Yu [2,3,†], Ji Won Ha [2], Seoung Rak Lee [2], Bum Soo Lee [2], Jin-Chul Kim [4], Jung Kyu Kim [5], Ki Sung Kang [1,*] and Ki Hyun Kim [2,*]

[1]College of Korean Medicine, Gachon University, Seongnam 13120, Korea

[2]School of Pharmacy, Sungkyunkwan University, Suwon 16419, Korea

[3]Department of Integrative Biological Sciences and Industry, Sejong University, Seoul 05006, Korea

[4]KIST Gangneung Institute of Natural products, Natural Product Informatics Research Center, Gangneung 25451, Korea

[5]School of Chemical Engineering, Sungkyunkwan University, Suwon, Gyeonggi-do, 16419, Republic of Korea

*Correspondence: kkang@gachon.ac.kr (K.S.K.); khkim83@skku.edu (K.H.K.); Tel.: +82-31-750-5402 (K.S.K.); +82-31-290-7700 (K.H.K.)

†These authors contributed equally to this study.

Supporting Information Contents:

Figure S1. The HR-ESIMS data of **1**……S3
Figure S2. The ^{1}H NMR spectrum of **1** (CD_3OD, 850 MHz)……S4
Figure S3. The ^{13}C NMR spectrum of **1** (CD_3OD, 212.5 MHz)……S5
Figure S4. The ^{1}H-^{1}H COSY spectrum of **1** (CD_3OD)……S6
Figure S5. The HSQC spectrum of **1** (CD_3OD)……S7
Figure S6. The HMBC spectrum of **1** (CD_3OD)……S8
Figure S7. The ROESY spectrum of **1** (CD_3OD)……S9
Figure S8. The HR-ESIMS data of **1a**……S10
Figure S9. The ^{1}H NMR spectrum of **1a** (CD_3OD, 850 MHz)……S11
Figure S10. The ^{13}C NMR spectrum of **1a** (CD_3OD, 212.5 MHz)……S12
Figure S11. The ^{1}H-^{1}H COSY spectrum of **1a** (CD_3OD)……S13
Figure S12. The HSQC spectrum of **1a** (CD_3OD)……S14
Figure S13. The HMBC spectrum of **1a** (CD_3OD)……S15
Figure S14. The ROESY spectrum of **1a** (CD_3OD)……S16

General experimental procedure……S17
Table S1. ^{1}H (850 MHz) and ^{13}C NMR (212.5 MHz) data for compounds **1** and **1a** in CD_3OD (δ ppm)……S19

Figure S1. The HR-ESIMS data of **1**

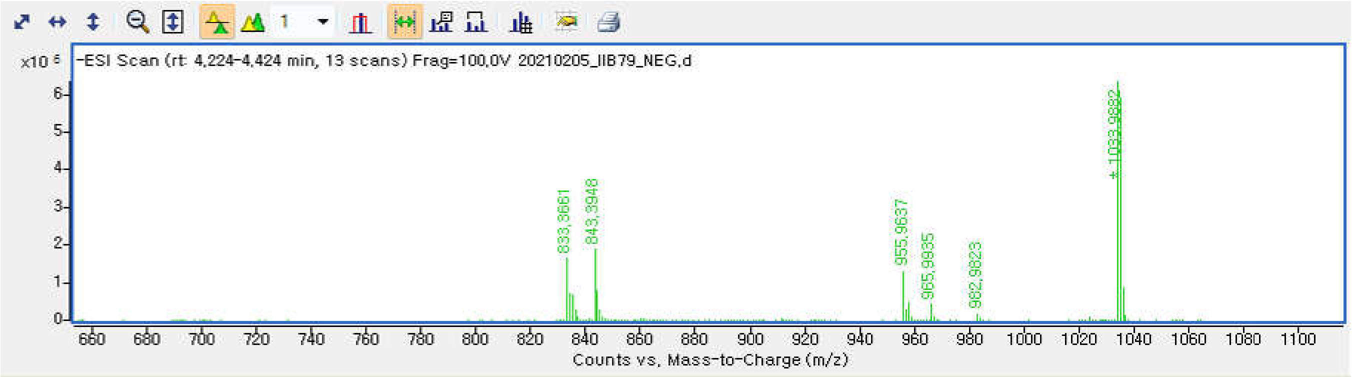

Figure S2. The ^{1}H NMR spectrum of **1** (CD_3OD, 850 MHz)

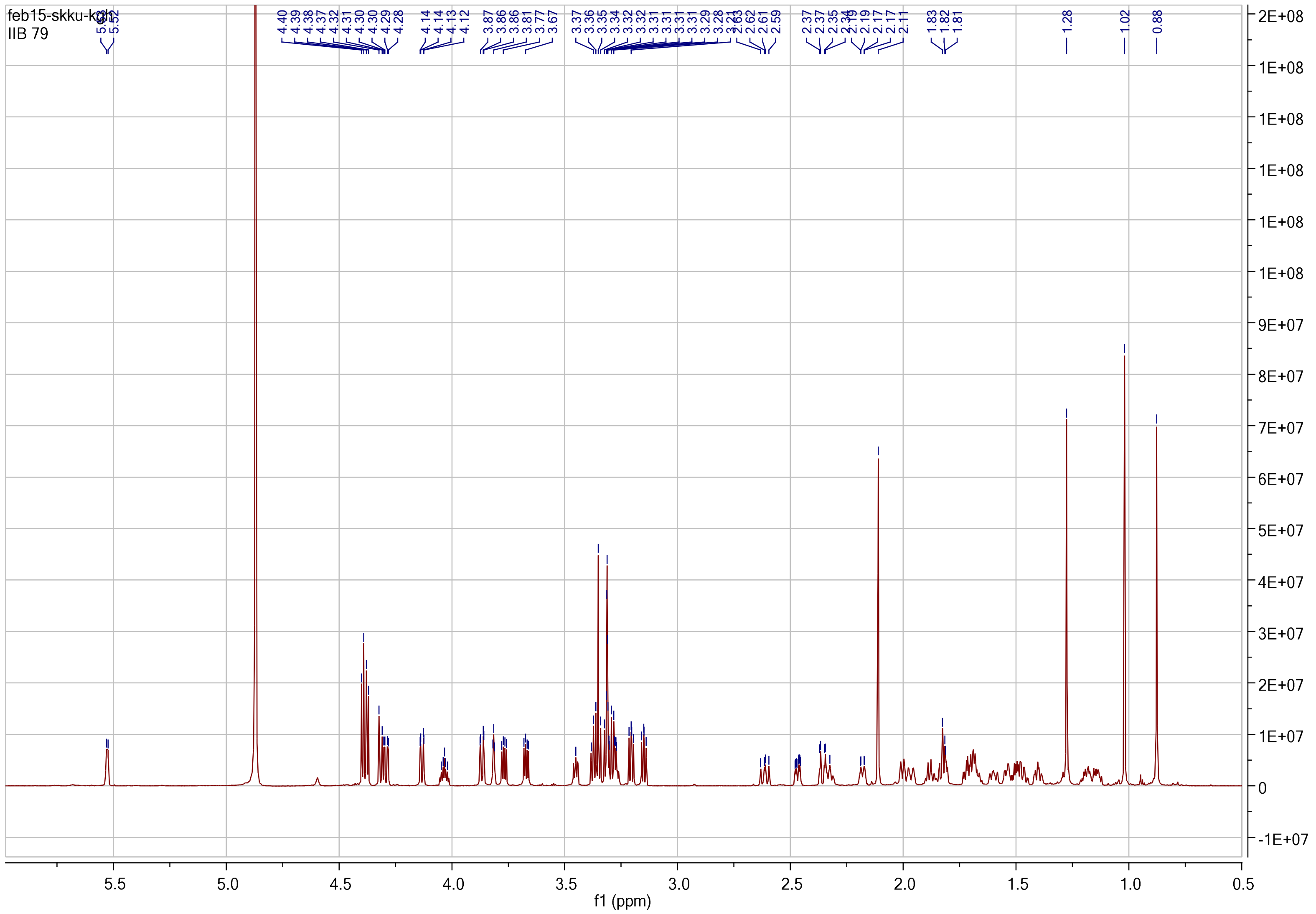

Figure S3. The ^{13}C NMR spectrum of **1** (CD_3OD, 212.5 MHz)

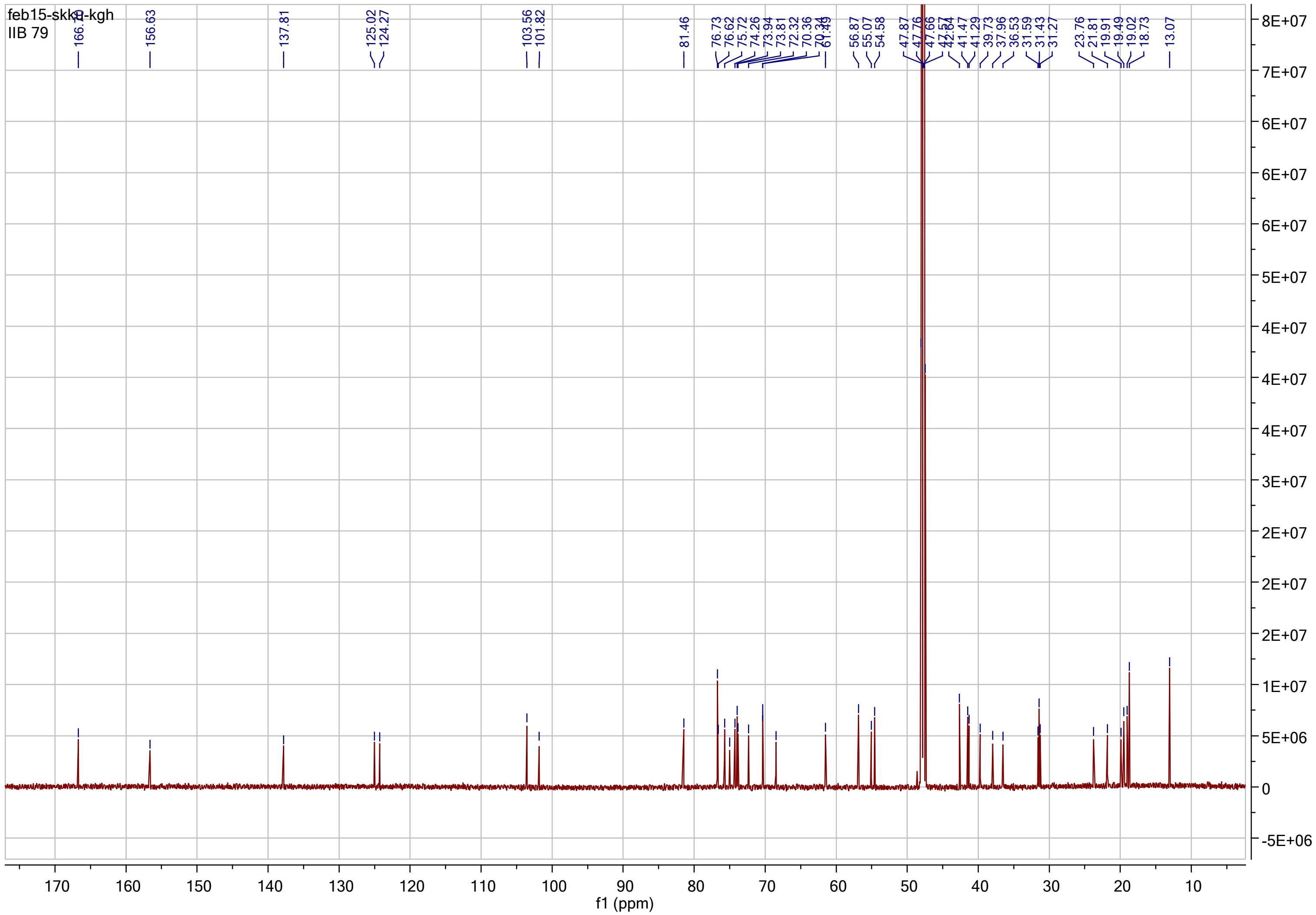

Figure S4. The ¹H-¹H COSY spectrum of **1** (CD_3OD)

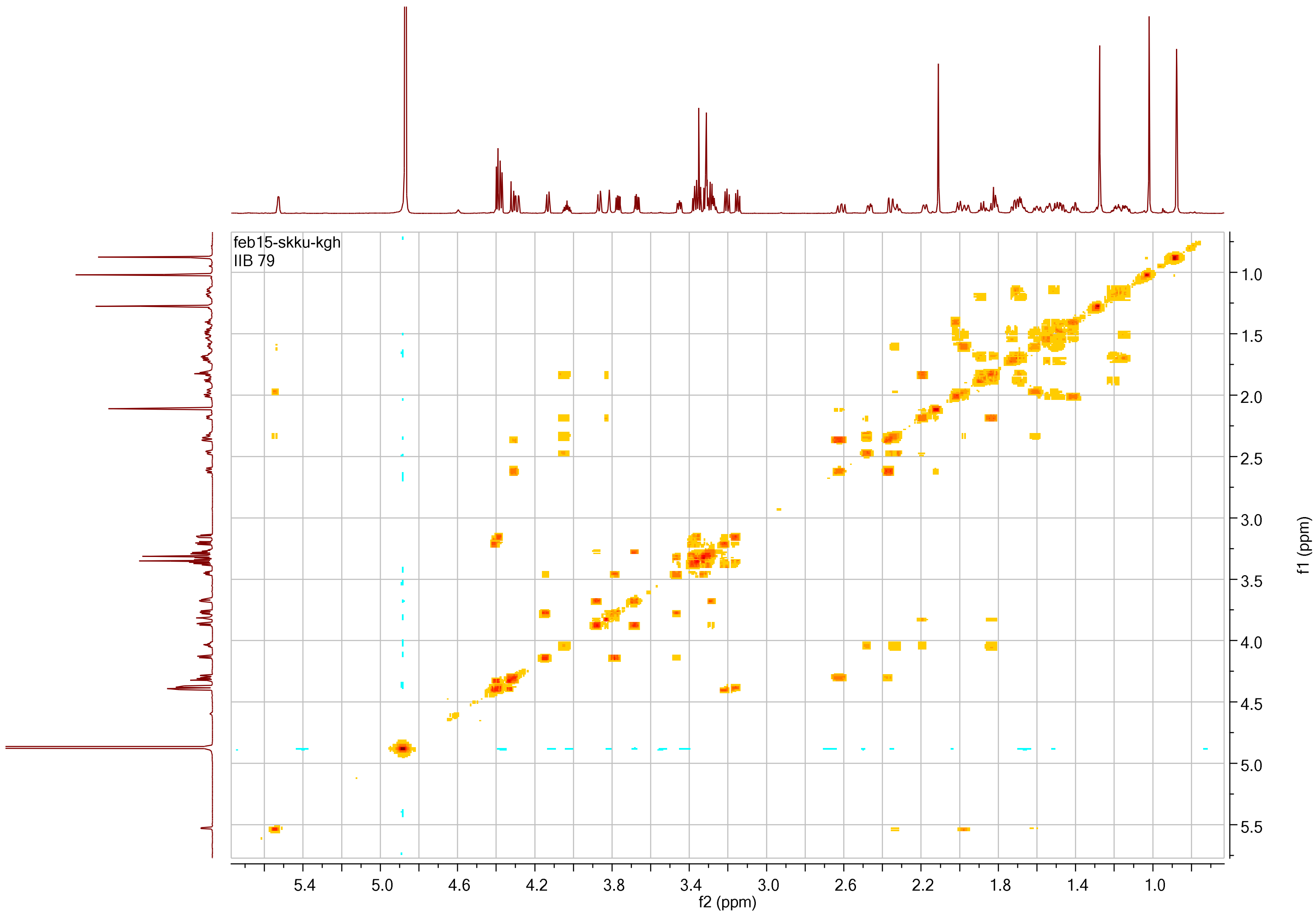

Figure S5. The HSQC spectrum of **1** (CD_3OD)

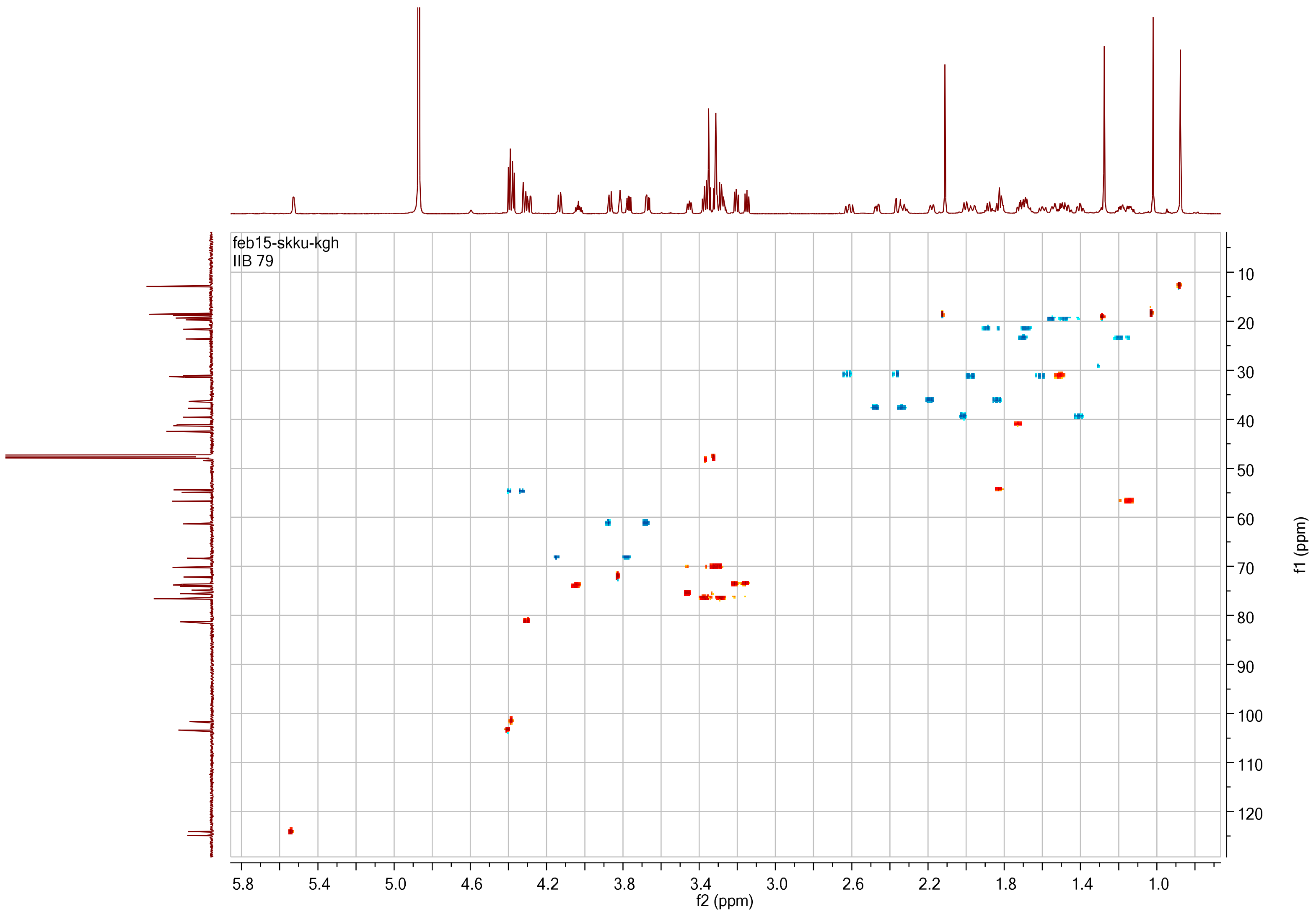

Figure S6. The HMBC spectrum of **1** (CD_3OD)

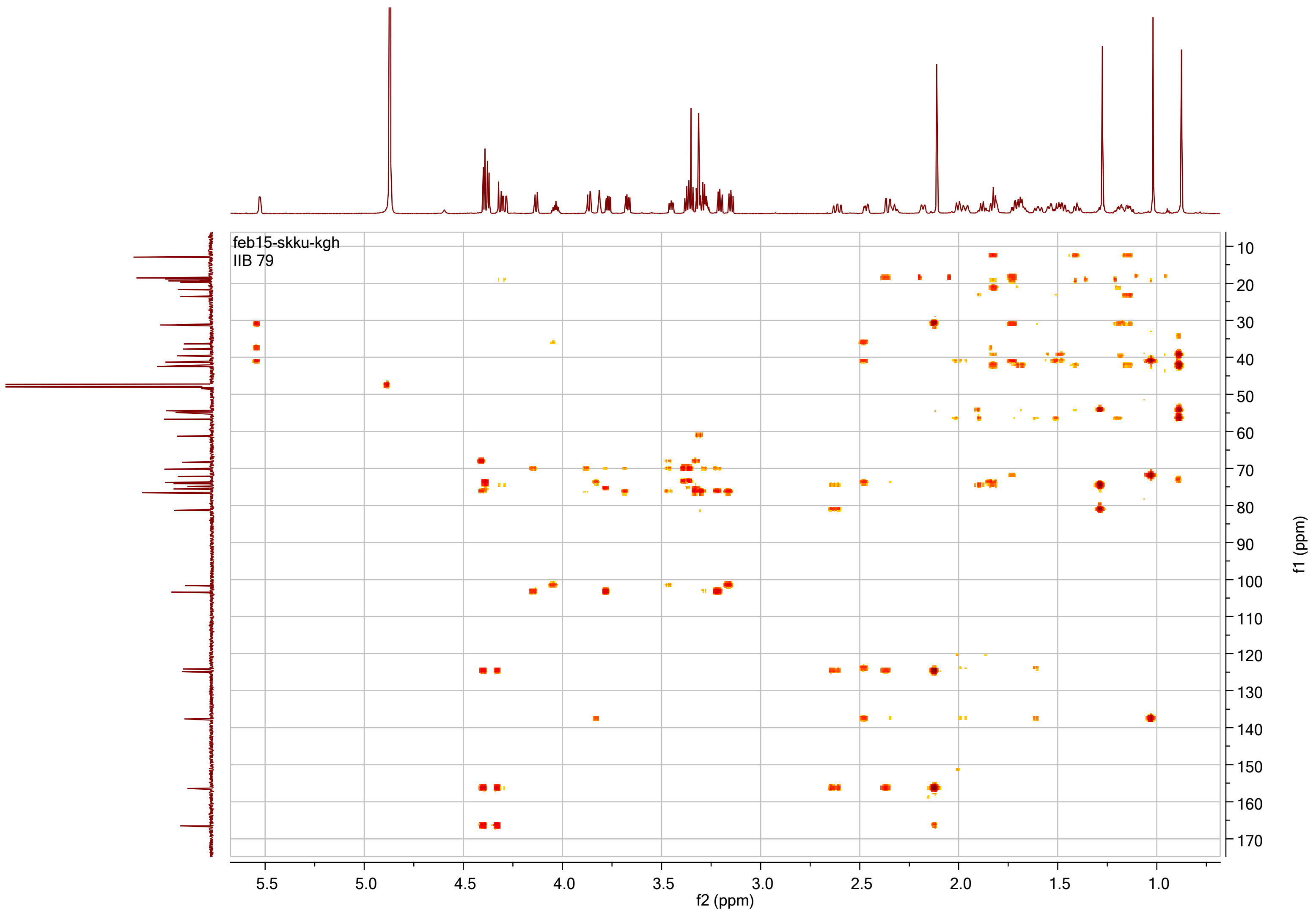

Figure S7. The ROESY spectrum of **1** (CD_3OD)

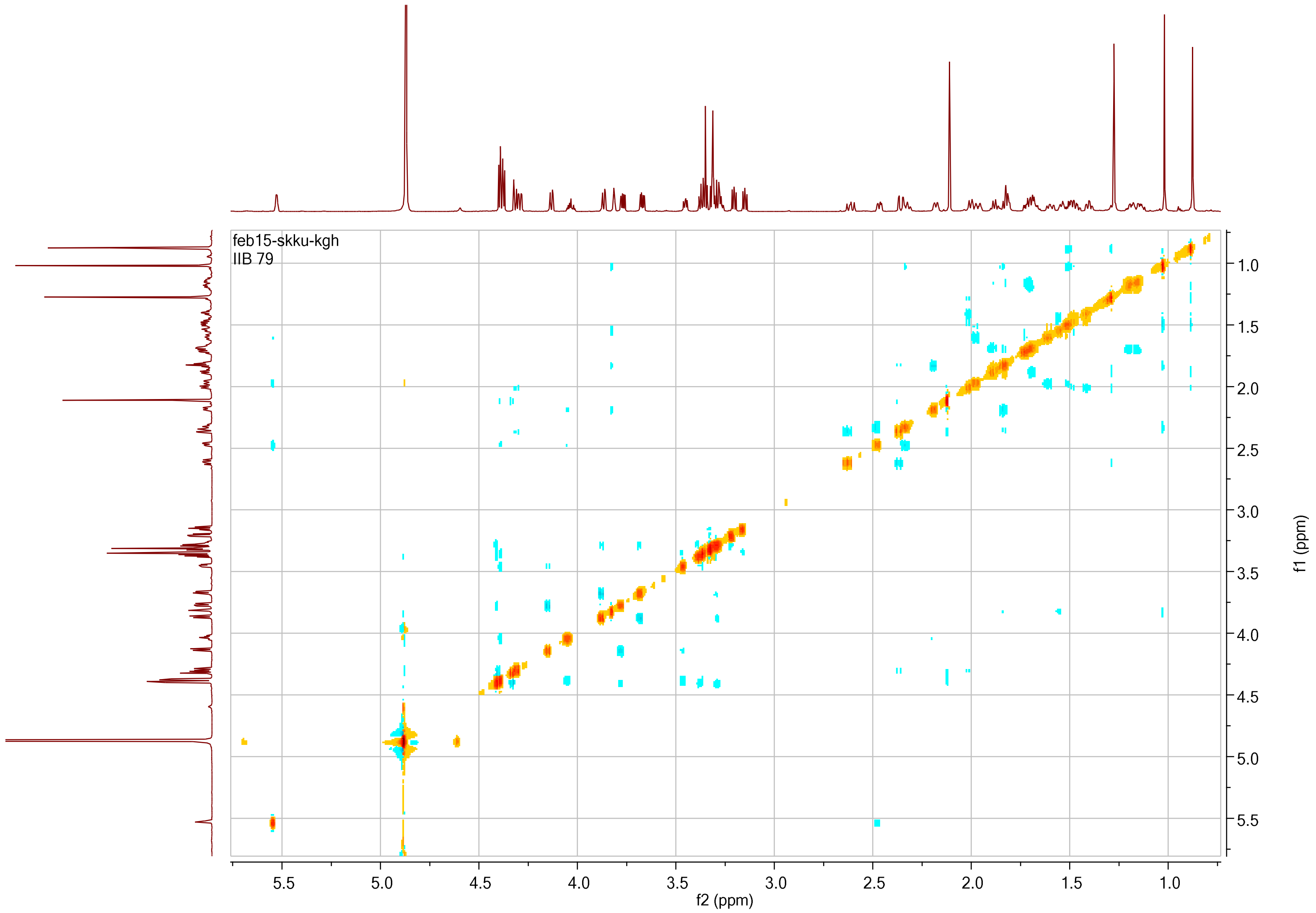

Figure S8. The HR-ESIMS data of **1a**

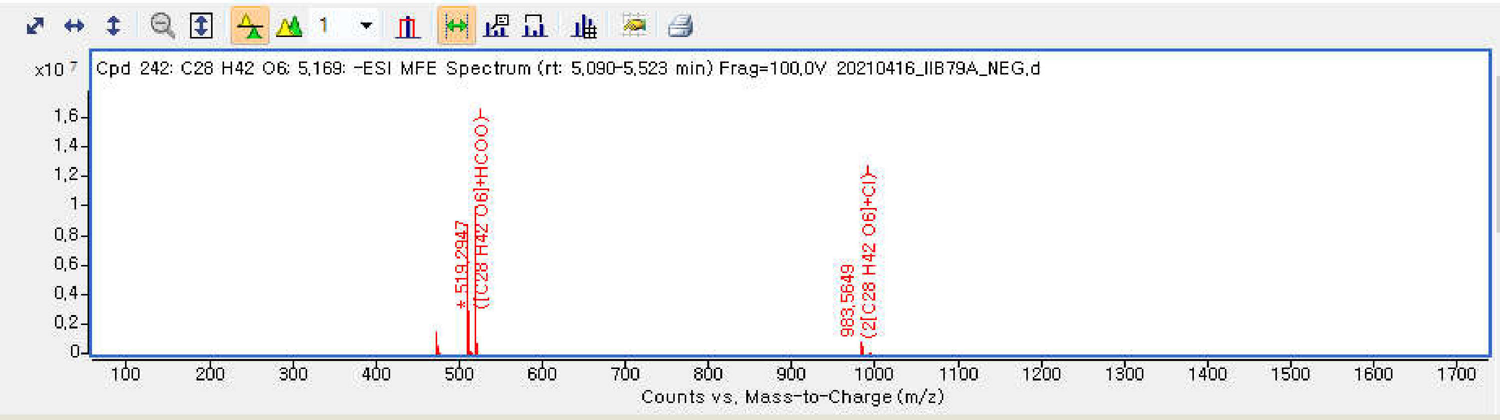

Figure S9. The ^{1}H NMR spectrum of **1a** (CD_3OD, 850 MHz)

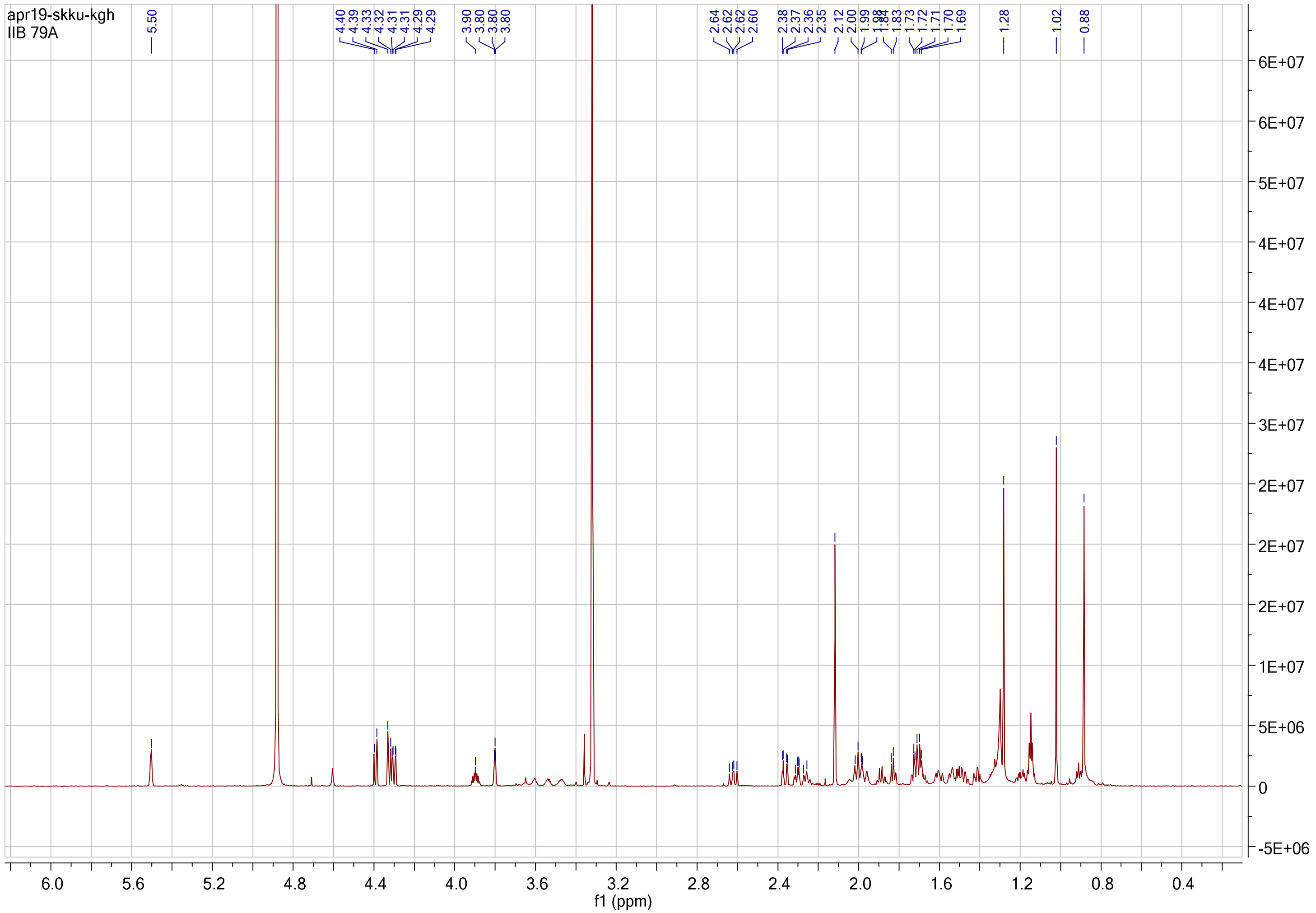

Figure S10. The ^{13}C NMR spectrum of **1a** (CD_3OD, 212.5 MHz)

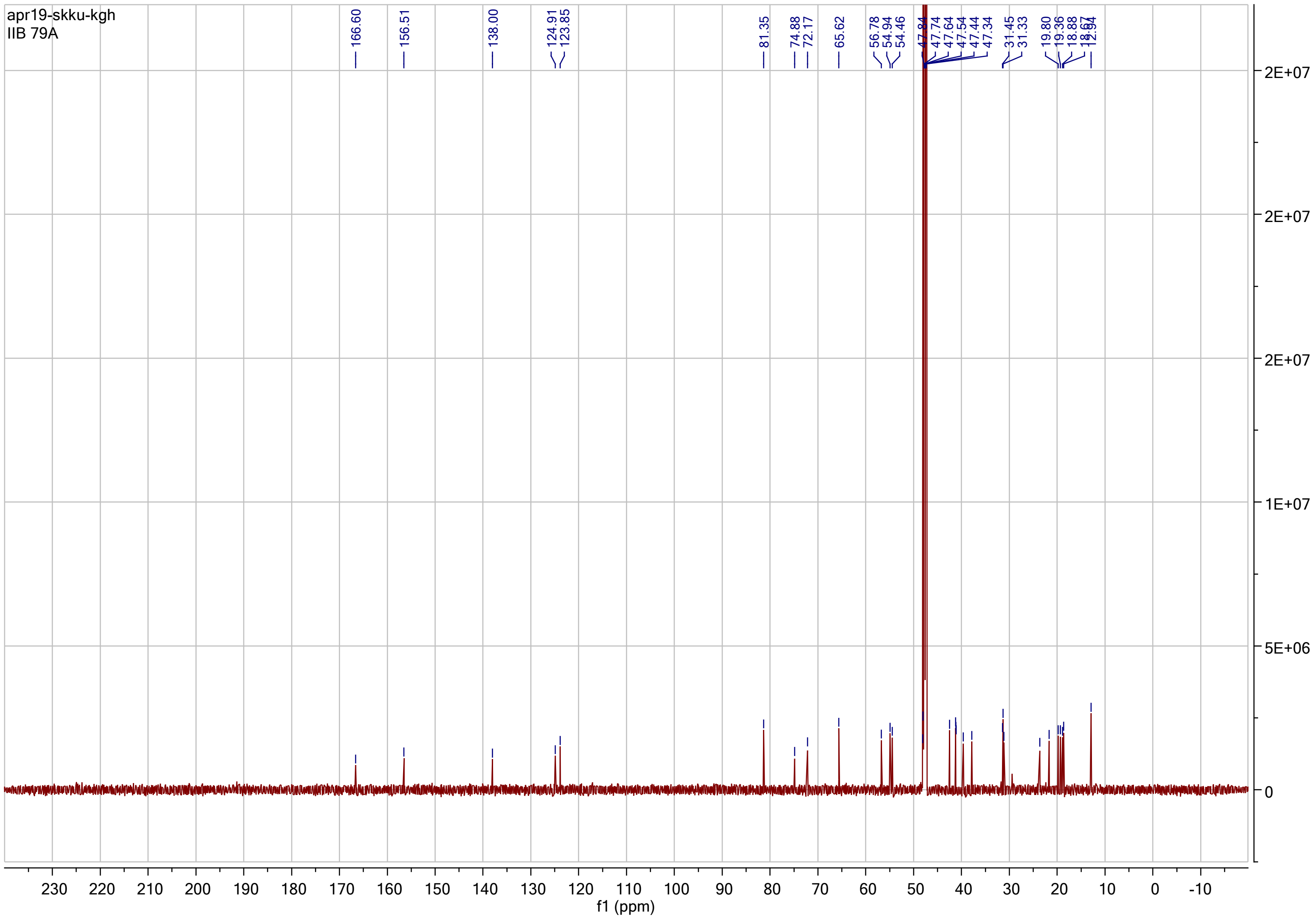

Figure S11. The ^{1}H-^{1}H COSY spectrum of **1a** (CD_3OD)

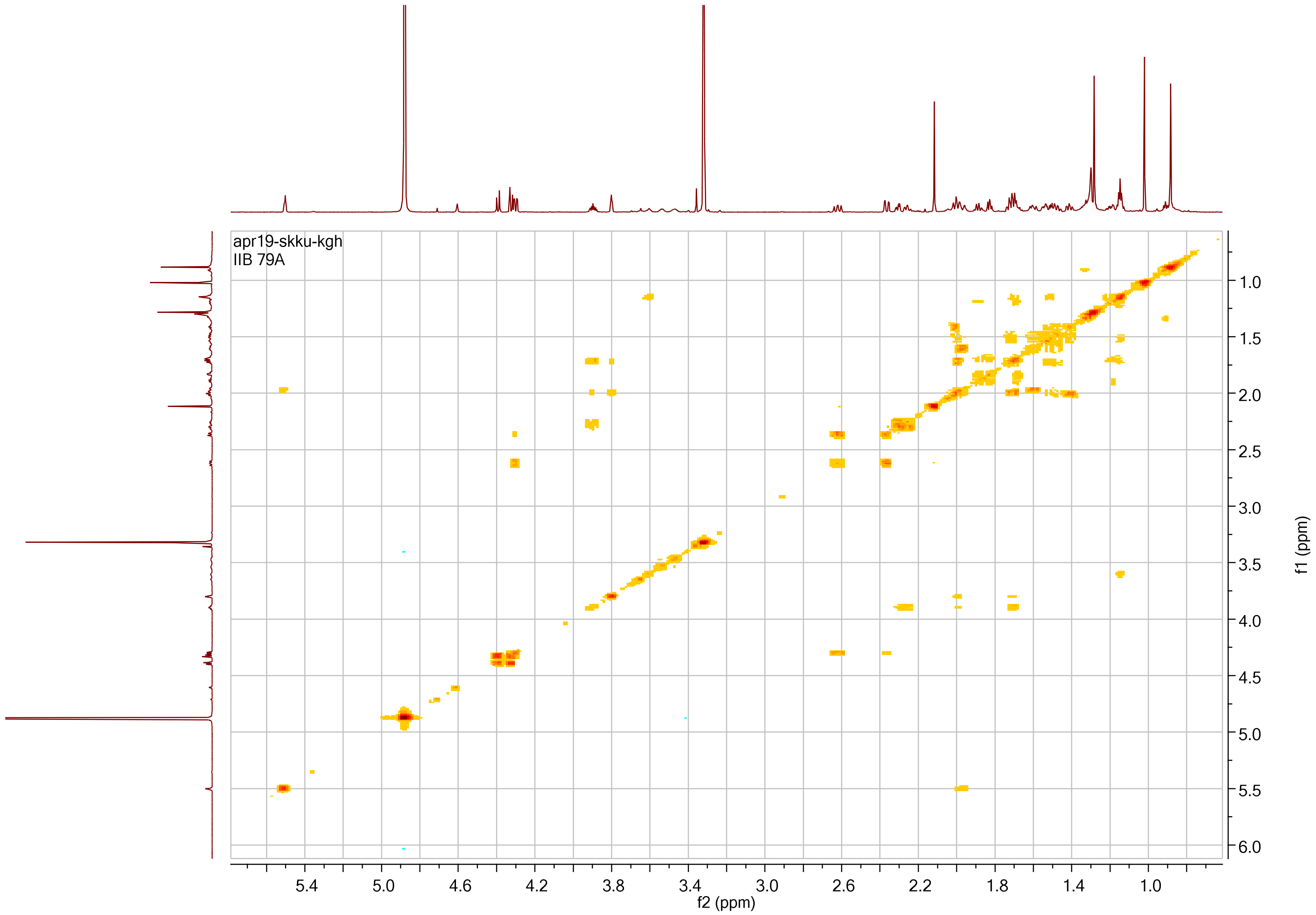

Figure S12. The HSQC spectrum of **1a** (CD_3OD)

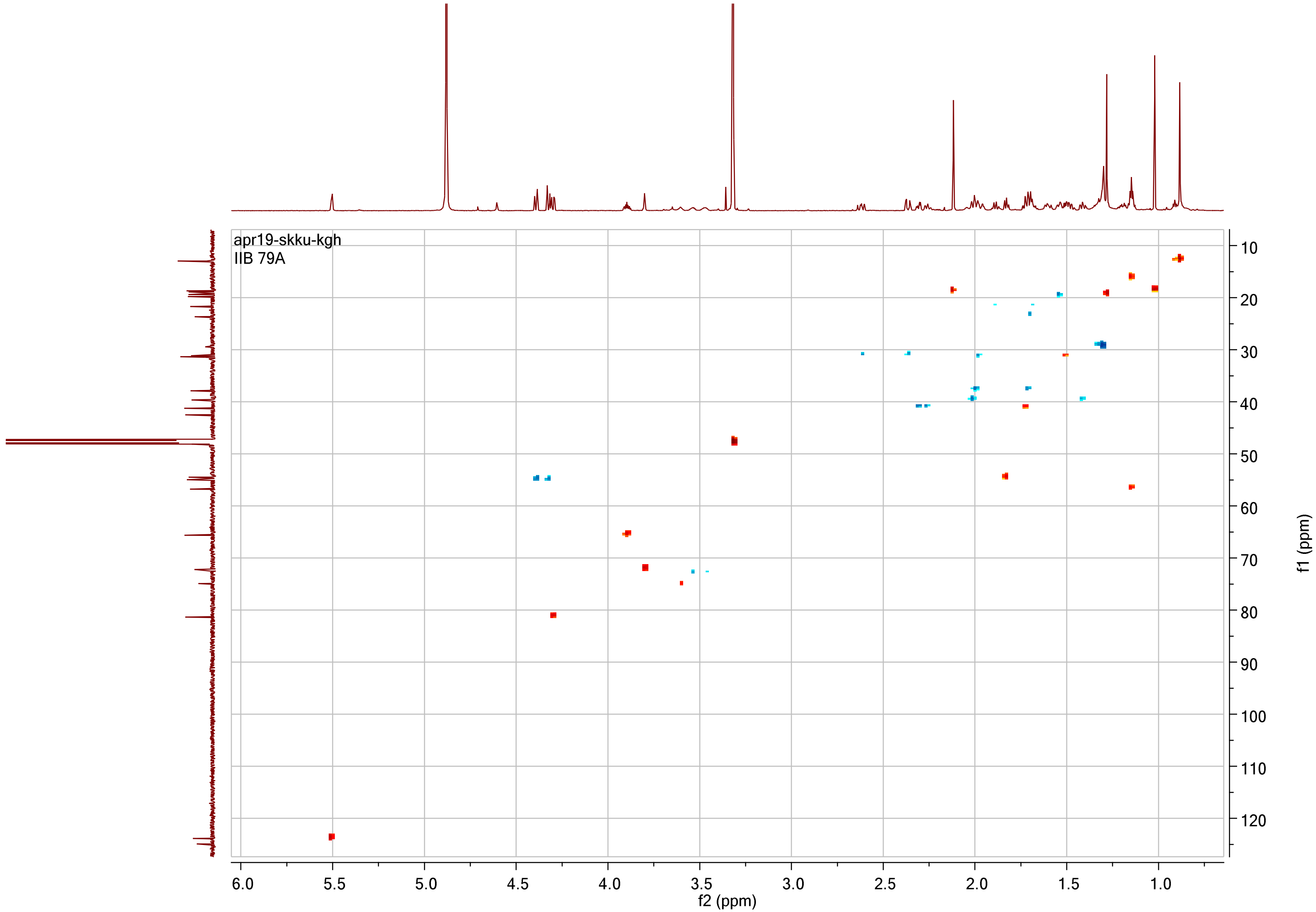

Figure S13. The HMBC spectrum of **1a** (CD_3OD)

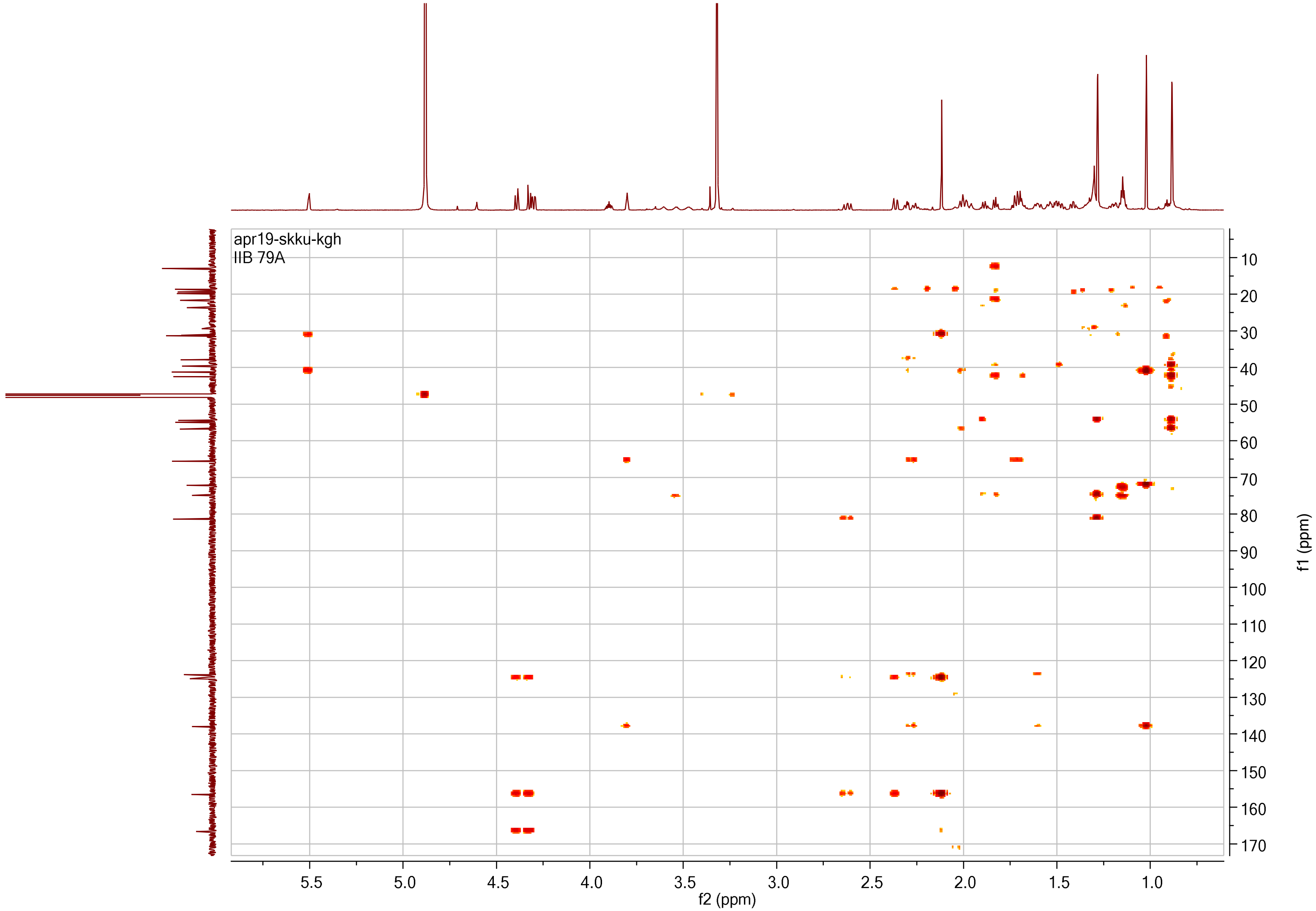

Figure S14. The ROESY spectrum of **1a** (CD_3OD)

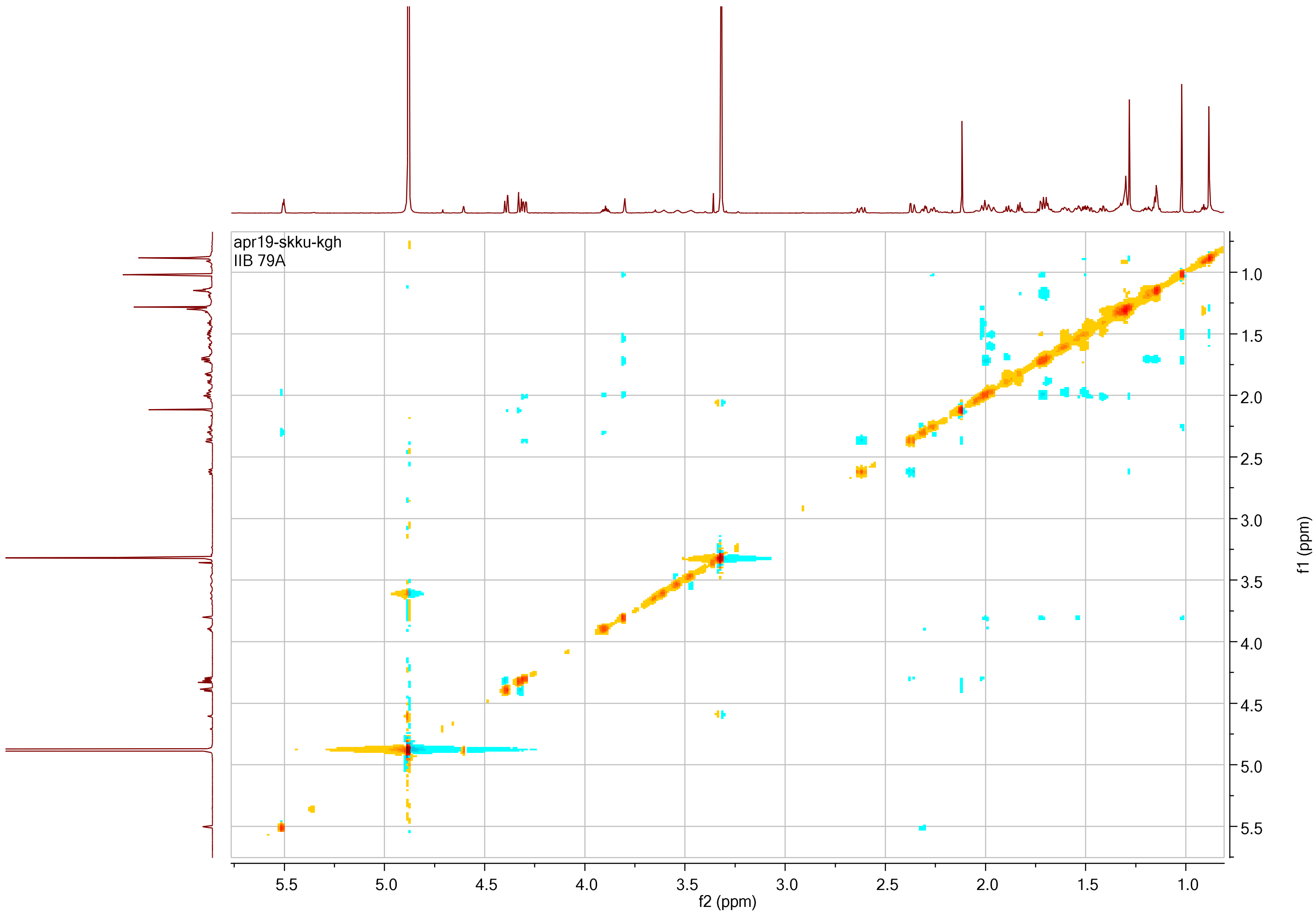

General experimental procedure

Optical rotations were measured using a JASCO P-2000 polarimeter (JASCO, Easton, MD, USA). Ultraviolet (UV) spectra were acquired on an Agilent 8453 UV-visible spectrophotometer (Agilent Technologies, Santa Clara, CA, USA). Electronic circular dichroism (ECD) spectra were measured on a JASCO J-1500 spectropolarimeter (JASCO). Infrared (IR) spectra were recorded with a Bruker IFS-66/S FT-IR spectrometer (Bruker, Karlsruhe, Germany). Nuclear magnetic resonance (NMR) spectra were recorded with a Bruker AVANCE III HD 800 NMR spectrometer with a 5 mm TCI CryoProbe operating at 850 MHz (^{1}H) and 212.5 MHz (^{13}C), with chemical shifts given in ppm (δ) for 1H and 13C NMR analyses. All HRESIMS data were obtained with a Waters Xevo G2 QTOF mass spectrometer and Synapt G2 HDMS quadrupole time-of-flight (TOF) mass spectrometer (Waters). Preparative high-performance liquid chromatography (HPLC) was performed using a Waters 1525 Binary HPLC pump with a Waters 996 Photodiode Array Detector (Waters Corporation, Milford, MA, USA) and an Agilent Eclipse C18 column (250 × 21.2 mm, 5 µm; flow rate: 5 mL/min; Agilent Technologies). Semi-preparative HPLC was performed using a Shimadzu Prominence HPLC System with SPD-20A/20AV Series Prominence HPLC UV-Vis detectors (Shimadzu, Tokyo, Japan) and a Phenomenex Luna C18 column (250 × 10 mm, 5 µm; flow rate: 2 mL/min; Phenomenex, Torrance, CA, USA). LC/MS analysis was performed on an Agilent 1200 Series HPLC system equipped with a diode array detector and 6130 Series ESI mass spectrometer using an analytical Kinetex C18 100 Å column (100 × 2.1 mm, 5 µm; flow rate: 0.3 mL/min; Phenomenex). Silica gel 60 (230–400 mesh; Merck, Darmstadt, Germany) and RP-C18 silica gel (Merck, 230–400 mesh) were used for column chromatography. The packing material for molecular sieve column chromatography was Sephadex LH-20 (Pharmacia, Uppsala, Sweden). Thin-layer chromatography (TLC) was performed with precoated silica gel F254 plates and RP-C18 F254s plates (Merck) and spots were detected under UV light or by heating after spraying with anisaldehyde-sulfuric acid.

Plant material

One-year old roots of *W. somnifera* were purchased from Seong-geo-san Farm, Cheonan, Korea in October 2016, and the plant was identified by one of the authors (K. H. Kim). A voucher specimen of the material (IDG-2016) was deposited in the herbarium of the School of Pharmacy, Sungkyunkwan University, Suwon, Korea.

Extraction and separation of the compounds

Dried roots of *W. somnifera* (1.28 kg) were extracted using 80% aqueous MeOH (3.0 L) for 3 days under reflux, performed three times, and filtered at room temperature. The filtrate was concentrated using a rotavapor to obtain the MeOH extract (189.6 g), suspended in water (700

mL) and partitioned with 700 mL of four solvents: Hx, MC, EA, and *n*-BuOH. The four fractions were obtained in the following order: Hx (3.4 g), MC (4.5 g), EtOAc (2.0 g), and *n*-BuOH-soluble (18.6 g) fractions. These four fractions, obtained from solvent partitioning, were examined for cytotoxic activity and underwent LC/MS analysis. The *n*-BuOH-soluble fraction showed the highest cytotoxic activity against HepG2. In addition, LC/MS analysis of the active fraction revealed the presence of withanolide glycosides based on detected peaks exhibiting the UV pattern (λ_{max} 200–230 nm) similar to reported withanolides [15] and molecular ion peaks ranging from *m/z* 780–820.

The *n*-BuOH-soluble fraction (18.6 g) was subjected to silica gel open column chromatography (150 g, eluted with CH_2Cl_2/MeOH [30:1 → 1:1]), to afford six fractions (B1–B6). Fraction B4 (0.9 g) was subjected to Sephadex LH-20 open column chromatography with CH_2Cl_2/MeOH (2:8) to yield six subfractions (B4a–B4f). Subfraction B4b (285 mg) was separated by Sephadex LH-20 open column chromatography with MeOH/H_2O (4:6 → 10:0) to yield four subfractions (B4b1–B4b4). Subfraction B4b3 (37 mg) was further purified by semi-preparative HPLC (58% MeOH) to yield compound **2** (t_R 25.3 min, 4.6 mg). Fraction B6 (340 mg) was subjected to reverse-phase (RP) silica gel open column chromatography with MeOH/H_2O (4:6 → 10:0), yielding four subfractions (B6a-B6d), and subfraction B6c (150 mg) was subjected to preparative HPLC (65% MeOH → 80% MeOH, gradient solvent system) to yield three subfractions (B6c1-B6c3). Subfraction B6c3 (60 mg) was separated by semi-preparative HPLC with 49% MeOH to obtain compound **3** (t_R 60.0, 10.2 mg). Subfraction B6d (170 mg) was fractionated using RP silica gel open column chromatography (40% MeOH → 100% MeOH, gradient solvent system) to obtain five subfractions (B6d1-B6d5). The subfraction B6d4 (16 mg) was purified using semi-preparative HPLC (47% MeOH) to yield compound **1** (t_R 61.0, 5.7 mg). Finally, the subfraction B6d5 (63 mg) was purified via semi-preparative HPLC using 53% MeOH to isolate compounds **4** (t_R 31.0, 5.8 mg) and **5** (t_R 35.2, 15.7 mg).

Absolute configuration determination of the sugar moieties of compound 1

The aqueous layer was evaporated under a vacuum evaporator and dissolved in anhydrous pyridine (0.5 mL) with the addition of *L*-cysteine methyl ester hydrochloride (1.0 mg). After the reaction mixture was heated at 60°C for 1 h, *o*-tolyl isothiocyanate (50 µL) was added and the mixture was kept at 60°C for 1 h. The reaction product was evaporated under a vacuum evaporator and dissolved in MeOH. After then, the dissolved reaction product was directly analyzed by LC/MS [MeOH/H_2O,1:9 → 7:3 gradient system (0–30 min), 100% MeOH (31–41 min), 0% MeOH (42–52 min); flow rate of 0.3 mL/min] using analytical Kinetex C_{18} 100 Å column (100 mm × 2.1 mm i.d., 5 µm). The sugar moieties from **1** were identified as D-glucopyranoses, based on a comparison with the retention time of an authentic sample, D-glucopyranose (t_R 18.7 min) in the LC/MS analysis.

Table S1. ^{1}H (850 MHz) and ^{13}C NMR (212.5 MHz) data for compounds **1** and **1a** in CD_3OD (δ ppm).[a.]

Position	**1**		**1a**	
	**δ$_H$ (*J* in Hz)**	**δ$_C$**	**δ$_H$ (*J* in Hz)**	**δ$_C$**
1	3.81 dd (1.5, 1.5)	72.2 d	3.79 dd (1.5, 1.5)	72.1 d
2α	2.19 ddd (13.0, 5.5, 1.5)	36.4 t	2.01 ddd (13.0, 5.5, 1.5)	37.7 t
2β	1.83 ddd (13.0, 12.5, 1.5)		1.73 ddd (13.0, 12.5, 1.5)	
3	4.03 dddd (12.5, 12.5, 5.5, 5.5)	74.1 d	3.89 dddd (12.5, 12.5, 5.5, 5.5)	65.7 d
4α	2.47 dd (13.0, 5.5)	37.8 t	2.30 dd (13.0, 5.5)	40.9 t
4β	2.32 dd (13.0, 12.5)		2.25 dd (13.0, 12.5)	
5		137.7 s		138.0 s
6	5.53 d (5.5)	124.2 d	5.40 d (4.5)	123.8 d
7α	1.98 m	31.3 t	1.97 m	31.1 t
7β	1.61 m		1.60 m	
8	1.51 m	31.2 d	1.51 m	31.2 d
9	1.73 m	41.2 d	1.73 m	41.3 d
10		41.1 s		41.1 s
11α	1.55 m	19.5 t	1.53 m	19.7 t
11β	1.49 m		1.48 m	
12α	1.41 m	39.6 t	1.39 m	39.4 t
12β	2.01 m		2.01 m	
13		42.5 s		42.3 s
14	1.14 m	56.6 d	1.14 m	56.6 d
15α	1.70 m	23.5 t	1.88 m	21.5 t
15β	1.20 m		1.67 m	
16α	1.70 m	21.6 t	1.18 m	23.6 t
16β	1.90 m		1.70 m	
17	1.82 m	54.4 d	1.82 m	54.1 d
18	0.88 s	13.1 q	0.89 s	12.8 q
19	1.02 s	18.5 q	1.02 s	18.6 q
20		73.3 s		74.6 s

21	1.27 s	19.5 q	1.28 s	19.3 q
22	4.29 dd (13.0, 3.5)	81.4 d	4.30 dd (13.0, 3.5)	81.2 d
23α	2.36 dd (18.0, 3.5)	31.1 t	2.35 dd (18.0, 3.5)	31.0 t
23β	2.61 dd (18.0, 13.5)		2.63 dd (18.0, 13.5)	
24		156.5 s		156.7 s
25		125.1 s		124.8 s
26		166.7 s		166.6 s
27	4.40 d (12.0); 4.32 d (12.0)	54.8 t	4.39 d (12.0); 4.32 d (12.0)	54.6 t
28	2.11 s	19.1 q	2.11 s	19.0 q
1’	4.37 d (8.0)	101.7 d		
2’	3.16 dd (9.0, 8.0)	73.8 d		
3’	3.37 overlap	76.5 d		
4’	3.28 overlap	70.3 d		
5’	3.45 overlap	75.7 d		
6’	4.13 dd (12.0, 2.0); 3.79 dd (12.0, 6.0)	68.4 t		
1”	4.39 d (8.0)	103.4 d		
2”	3.20 (9.0, 8.0)	73.8 d		
3”	3.36 overlap	76.4 d		
4”	3.31 overlap	70.0 d		
5”	3.28 overlap	76.5 d		
6”	3.88 dd (12.0, 2.0); 3.69 dd (12.0, 5.5)	61.2 t		

[a]*J* values are in parentheses (shown in Hz); ^{13}C NMR assignments are based on HSQC and HMBC experiments. ^{13}C NMR, Carbon-13 (C13) nuclear magnetic resonance; HMBC, heteronuclear multiple bond correlation; HSQC, heteronuclear single quantum correlation.
